# microRNA-124 inhibits bone metastasis of breast cancer by repressing Interleukin-11

**DOI:** 10.1186/s12943-017-0746-0

**Published:** 2018-01-17

**Authors:** Wei-Luo Cai, Wen-Ding Huang, Bo Li, Tian-Rui Chen, Zhen-Xi Li, Cheng-Long Zhao, Heng-Yu Li, Yan-Mei Wu, Wang-Jun Yan, Jian-Ru Xiao

**Affiliations:** 1 Department of Musculoskeletal Tumor, Fudan University Shanghai Cancer Center, Department of Oncology, Shanghai Medical College, Fudan University, 270 Dong An Road, Shanghai, 200032 People’s Republic of China; 2 Spine Tumor Center, Changzheng Hospital, Second Military Medical University, 415 Feng Yang Road, Shanghai, 200003 People’s Republic of China; 3 Department of Breast and Thyroid Surgery, General Surgery, Changhai Hospital, Second Military Medical University, Shanghai, 200433 China

**Keywords:** Breast cancer, Bone metastasis, miR-124, IL-11, Bone microenvironment

## Abstract

**Background:**

Most patients with breast cancer in advanced stages of the disease suffer from bone metastases which lead to fractures and nerve compression syndromes. microRNA dysregulation is an important event in the metastases of breast cancer to bone. microRNA-124 (miR-124) has been proved to inhibit cancer progression, whereas its effect on bone metastases of breast cancer has not been reported. Therefore, this study aimed to investigate the role and underlying mechanism of miR-124 in bone metastases of breast cancer.

**Methods:**

In situ hybridization (ISH) was used to detect the expression of miR-124 in breast cancer tissues and bone metastatic tissues. Ventricle injection model was constructed to explore the effect of miR-124 on bone metastasis in vivo. The function of cancer cell derived miR-124 in the differentiation of osteoclast progenitor cells was verified in vitro. Dual-luciferase reporter assay was conducted to confirm Interleukin-11 (IL-11) as a miR-124 target. The involvement of miR-124/IL-11 in the prognosis of breast cancer patients with bone metastasis was determined by Kaplan-Meier analysis.

**Results:**

Herein, we found that miR-124 was significantly reduced in metastatic bone tissues from breast cancers. Down-regulation of miR-124 was associated with aggressive clinical characteristics and shorter bone metastasis-free survival and overall survival. Restoration of miR-124 suppressed, while inhibition of miR-124 promoted the bone metastasis of breast cancer cells in vivo. At the cellular level, gain of function and loss-of function assays indicated that cancer cell-derived miR-124 inhibited the survival and differentiation of osteoclast progenitor cells. At the molecular level, we demonstrated that IL-11 partially mediated osteoclastogenesis suppression by miR-124 using in vitro and in vivo assays. Furthermore, IL-11 levels were inversely correlated with miR-124, and up-regulation IL-11 in bone metastases was associated with a poor prognosis.

**Conclusions:**

Thus, the identification of a dysregulated miR-124/IL-11 axis helps elucidate mechanisms of breast cancer metastases to bone, uncovers new prognostic markers, and facilitates the development of novel therapeutic targets to treat and even prevent bone metastases of breast cancer.

**Electronic supplementary material:**

The online version of this article (10.1186/s12943-017-0746-0) contains supplementary material, which is available to authorized users.

## Background

Breast cancer is one of the most common cancers and is the leading cause of cancer-related deaths among women worldwide [1]. In recent years, significant advances in early diagnosis and novel treatments for breast cancer have been achieved and have improved overall survival. However, metastasis remains the underlying cause of death for the majority of breast cancer patients and represents a large obstacle to reducing the mortality of advanced breast cancer [2]. Bone is the most common site for breast cancer metastases [3], and bone metastasis leads to skeletal-related events (SREs) including osteodynia, pathological fracture and spinal cord compression, which aggravate life quality and shorten overall survival of breast cancer patients [4].

Bone metastasis is an orchestrated process, with tumor cells exiting the primary site, disseminating to bone, and surviving in the bone microenvironment [5]. In this microenvironment, tumor cells have complicated interactions with osteoblasts, osteoclasts and bone stromal cells, which leads to osteogenesis/osteoclastogenesis imbalance, enabling breast cancer cells to form osteolytic lesions in bone and causing SREs [6]. This process is controlled by systemic and local signals as well as a number of cytokines, such as IL-11 [7]. IL-11 is a member of the Interleukin-6 (IL-6) family cytokines, which exert pleiotropic effects including stimulating hemopoiesis and thrombopoiesis, regulating macrophage differentiation, and conferring mucosal protection in the intestine [8]. In addition to its well-known roles as a hemopoietic growth factor, IL-11 functions as a prominent pro-tumorigenic cytokine in epithelial cancers such as breast cancer by activating the GP130-Janus kinase signaling cascade [9]. McCoy et al. [10] revealed that IL-11 produced by breast cancer cells augments osteoclastogenesis by sustaining the pool of osteoclast progenitor cells, suggesting a potential role for IL-11 in the bone metastasis of breast malignancies. Therefore, inhibiting IL-11 signaling might be a promising therapeutic opportunity to treat bone metastases from breast cancer. However, IL-11 inhibitors involved in breast tumor-stromal interactions have been poorly defined.

microRNAs (miRNAs) are a class of important posttranscriptional regulators that target key pathways involved in breast cancer metastasis and critical chemokines or cytokines in the bone microenvironment [11]. Therefore, many miRNAs have been found to be dysregulated in breast cancer and are versatile mediators of the complex interactions between cancer cells and the osseous microenvironment [12]. miR-124 was first cloned from mouse brain tissue [13], is the most abundant miRNA in the brain, and has reported functions in nervous system development [14]. Several nervous system diseases are related to miR-124 dysregulation [15]. In recent years, a growing number of studies have elucidated the role of miR-124 in the pathogenesis of malignant tumors, including hepatocellular carcinoma [16], cervical cancer [17], leukemia [18], gastric cancer [19], colorectal cancer [20], pancreatic cancer [21], prostate cancer [22], and, in particular, breast cancer [23–29]. All of these studies have identified miR-124 as a tumor suppressor [16–29]. Furthermore, some of them demonstrated that miR-124 could inhibit the proliferation, migration and invasion of breast cancer cells [23–28], indicating a critical role in breast cancer metastasis, although to date, far less is known about its effect on bone metastases from breast cancer.

In this study, we clarified the role of cancer cell-derived miR-124 in bone metastases from breast cancer and identified that perturbation of the miR-124/IL-11 regulatory axis contributes to the bone metastasis observed in breast cancer. These findings might provide novel therapeutic and diagnostic targets for bone metastases from breast cancer.

## Methods

### Cell culture

Human breast cancer cell lines (BT-549, MDA-MB-231, Hs578T, MDA-MB-468, MDA-MB-436, MCF7, T47D and BT-474) as well as RAW264.7 and MC3T3-E1 were obtained from ATCC. The strongly bone-metastatic MDA-MB-231-derived subline, MDA-MB-231-luc-D3H2LN, was purchased from Xenogen Corporation (Alameda, CA, USA). Bone marrow-derived macrophages (BMMs) were isolated from C57/BL6 mice as described previously [30]. All of the cell lines were tested using MycoAlert Mycoplasma Detection Kit (Lonza, Switzerland) according to the manufacturer’s instructions and were free of mycoplasma contamination.

### Clinical tissues

Primary breast cancer tissues and adjacent non-tumorous mammary tissues were obtained from patients who received breast cancer surgery and were histopathologically verified at Changhai hospital. Bone metastases from breast cancer were obtained from patients that received bone metastasis resection at Changzheng hospital (Shanghai, China). All of the subjects provided written informed consent. Ethical consent was granted by the Committees for Ethical Review of Research involving Human Subjects of Second Military Medical University (Shanghai, China).

### In situ hybridization (ISH)

Fluorescence ISH was used to detect miR-124 expression in tissue microarray slides containing 79 paired primary breast cancer tissues and adjacent non-tumorous mammary tissues as well as 34 bone metastases from breast cancer. ISH was performed using a miR-124 locked nucleic acid probe (5′-digoxigenin-GGCATTCACCGCGTGCCTTA-3′-digoxigenin) and the microRNA ISH Optimization Kit (Exiqon, Vedbaek, Denmark) according to the manufacturer’s instructions as described previously [31]. The signals were examined with a BX51 fluorescence microscope (Olympus) and quantified using the Aperio Spectrum® software with a pixel count algorithm.

### Construction of lentivirus

To construct the lenti-miR-124 plasmid (pLenO-DCE-Puro-miR-124), cDNA encoding pri-miR-124 was appended with EcoRI and BamHI sites and cloned into pLenO-DCE-Puro Vector (Bio-link, Shanghai, China). The backbone plasmid expressing Green Fluorescent Protein (GFP) was used as a negative control (NC). Lentiviruses were produced by four-plasmid cotransfection of 293 T cells with the packaging helper plasmid pRSV-Rev., pMDLg/pRRE, pMD2.G and pLenO-DCE (Transfer Vector). The lentivirus carrying miR-124 inhibitor and its negative control (NC) were constructed by Obio Technology (Shanghai, China) according to the method described previously [32]. TuD-miR-124 fragment containing two miR-124 binding sequence or its control fragment was synthesized and cloned into the plasmid pLKD-CMV-DsRed2-U6-shRNA digested with AgeI and EcoRI.

### Animal models

To investigate the effect of miR-124 on breast cancer cell survival in the bone microenvironment, luciferase-labeled MDA-MB-231 cells stably expressing miR-124 or NC were transplanted into female Balb/c nude mice via the intratibia route. To investigate the effect of miR-124 on the bone metastasis of breast cancer cells in vivo, luciferase-labeled MDA-MB-231 cells stably expressing miR-124 or NC, as well as luciferase-labeled MCF7 cells stably expressing miR-124 inhibitor or NC were inoculated into the left ventricle of nude mice. To explore if miR-124 can prevent bone metastasis, the left ventricle of nude mice were inoculated with luciferase-labeled MDA-MB-231 cells and then treated with injections of 10 nmol ago-miR-124 (miR400004422, RiboBio, Guangzhou, China) or NC (miR04201) via tail vein. Bone metastases were analyzed by X-ray and histopathologically confirmed with H&E staining. To determine the role of IL-11 in the suppression of bone metastasis by miR-124, MCF7 cells stably expressing miR-124 inhibitor or NC were firstly inoculated into the left ventricle of nude mice, and then 5 μg IL-11 neutralizing antibody or the control IgG were injected into the tail veins of the mice twice a week for up to 3 weeks in both groups. All experimental mice were monitored with an ex vivo imaging system (IVIS200, Caliper LS, Hopkinton, MA, USA). All of the mouse experiments were performed according to protocols reviewed and approved by the Institutional Animal Care and Use Committee at the Second Military Medical University.

### Transfection of miR-124 mimic or miR-124 inhibitor and collection of conditioned media

miR-124 mimic and negative control (NC) or miR-124 inhibitor and inhibitor NC were purchased from RiboBio (RibiBio, Guangzhou, China). MDA-MB-231 cells were transfected with miR-124 mimic and NC, and MCF7 cells were transfected with miR-124 inhibitor and inhibitor NC using Lipofectamine2000 (Invitrogen) according to the manufacturer’s instructions. Twenty-four hours after transfection, the media from MDA-MB-231 cells transfected with miR-124 mimic or NC and the media from MCF7 cells transfected with miR-124 inhibitor or inhibitor NC were collected as conditioned media.

### Proliferation and differentiation of BMMs

BMMs (5 × 10^3^ cells/well) were seeded into 96-well plate and incubated with M-CSF (10 ng/ml) and the receptor activator of nuclear factor-κB ligand (RANKL) (50 ng/ml) in the presence of 30% conditioned media, which was replaced every 48 h. The proliferation of BMM cells was analyzed using Cell Counting Kit-8 (Dojinodo, Shanghai, China) according to the manufacturer’s protocol. After 5–7 days, BMM differentiation was analyzed by tartrate resistant acid phosphatase (TRAP) staining and actin-ring formation assays.

### Construction of luciferase reporter plasmids and luciferase reporter assays

To construct the luciferase reporter plasmid encoding IL-11 3’untranslated regions (3′UTR), a 1562 bp fragment of the 3′UTR from human IL-11 was sub-cloned into the psiCHECK2 vector (Promega, Madison, WI, USA) using XhoI and NotI restriction sites. Mutation of the miR-124 binding site on the IL-11 3′UTR reporter vector was performed using overlap extension by PCR as described previously [33]. The luciferase reporter assays were performed with the Dual-Luciferase Reporter Assay (Promega, Madison, WI, USA) using a luminometer (Synergy™ 4 Hybrid Microplate Reader, BioTek, Winooski, VT, USA).

### Reagent

Human IL-11 neutralizing antibody (AF-218-NA), normal goat IgG control antibody (AB-108-C) and recombinant human IL-11 protein (218-IL-005) were purchased from R&D systems (Minneapolis, MN, USA).

### Statistics

All statistical analyses were performed using SPSS version 21.0 software. Statistical tests for data analysis included two-tailed Student’s t test, log-rank test, Mann–Whitney U test, Spearman correlation analysis, and Fisher’s exact test. A *P* value < 0.05 was considered statistically significant.

Additional details of methods are described in the Additional file 1.

## Results

### miR-124 is down-regulated in invasive breast cancer cells and human bone metastatic tissues

**Fig. 1:**
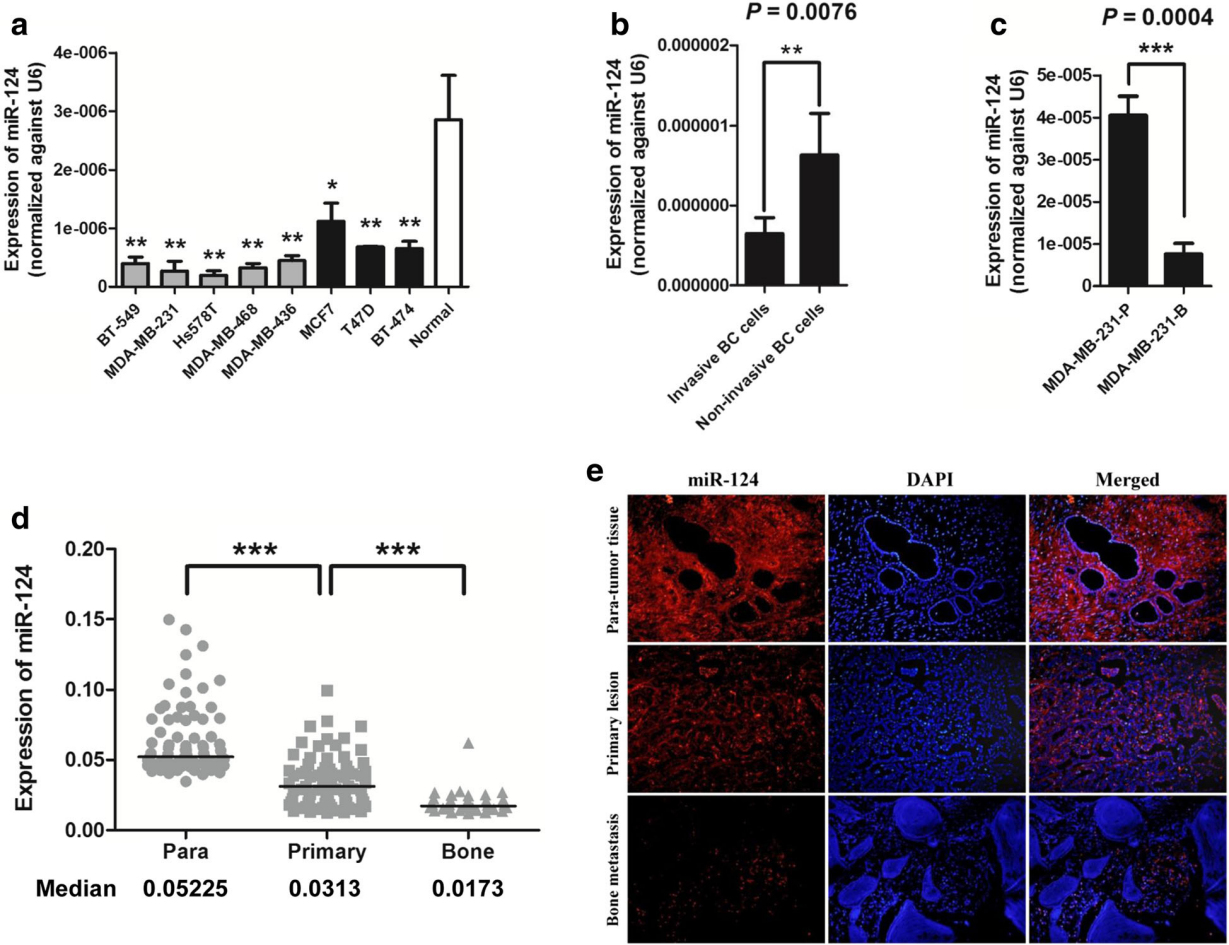
miR-124 is downregulated in metastatic cell lines and human tissues of breast cancer. **a** Real-time RT-PCR quantification of miR-124 expression in normal breast tissues (Normal) and eight kinds of breast cancer cells. Expression of miR-124 was normalized against an endogenous control U6. A Student t test was used to compare miR-124 expression in each individual cell with that in normal breast tissues. * *P* < 0.05, ** *P* < 0.01. **b** miR-124 expression was lower in invasive breast cancer cells, including BT-549, MDA-MB-231, Hs578T, MDA-MB-468 and MDA-MB-436, than in non-invasive breast cancer cells including MCF7, T47D and BT-474. **c** miR-124 expression was lower in MDA-MB-231 variant with high bone metastatic potential (MDA-MB-231-B) than in parental MDA-MB-231 cells (MDA-MB-231-P). **d** In situ microRNA hybridization (ISH) assay of miR-124 level in 79 pairs of primary breast cancer tissues (Primary) and para-tumor tissues (Para) as well as 34 bone metastasis tissues (bone) of breast cancer. Horizontal line indicates median value. ***P < 0.001 by Mann–Whitney U test. **e** Representative ISH images of miR-124 expression in para-tumor tissues, primary lesions and bone metastasis tissues at an original magnification of ×40

We examined miR-124 expression in normal human mammary tissue and a panel of human breast cancer cell lines with different invasive properties. miR-124 levels were significantly decreased relative to the control in all of the eight tested breast cancer cell lines (Fig. 1a). Furthermore, miR-124 expression was substantially lower in the invasive cell lines (BT-549, MDA-MB-231, Hs578T, MDA-MB-468 and MDA-MB-436) than in the non-invasive cell lines (MCF7, T47D and BT-474) (Fig. 1b). MDA-MB-231-luc-D3H2LN (MDA-MB-231-B) is a commonly used metastatic breast cancer cell line that has strong bone-metastatic characteristics. miR-124 expression was significantly down-regulated in MDA-MB-231-B compared to parental MDA-MB-231 cells (MDA-MB-231-P) (Fig. 1c). We also detected miR-124 expression in 79 paired primary breast cancer tissues and adjacent non-tumorous mammary tissues as well as 34 bone metastases from breast cancers using fluorescence ISH. miR-124 expression was substantially repressed in primary lesions compared to paired non-tumor tissues (median, 0.0313 and 0.05225, respectively; *P* < 0.001; Mann-Whitney’s U test) and was further reduced in metastatic bone tissues (median, 0.0173; *P* < 0.001; Mann-Whitney’s U test) (Fig. 1d, e).

### miR-124 suppresses survival of breast cancer cells in the bone microenvironment in mice

**Fig. 2:**
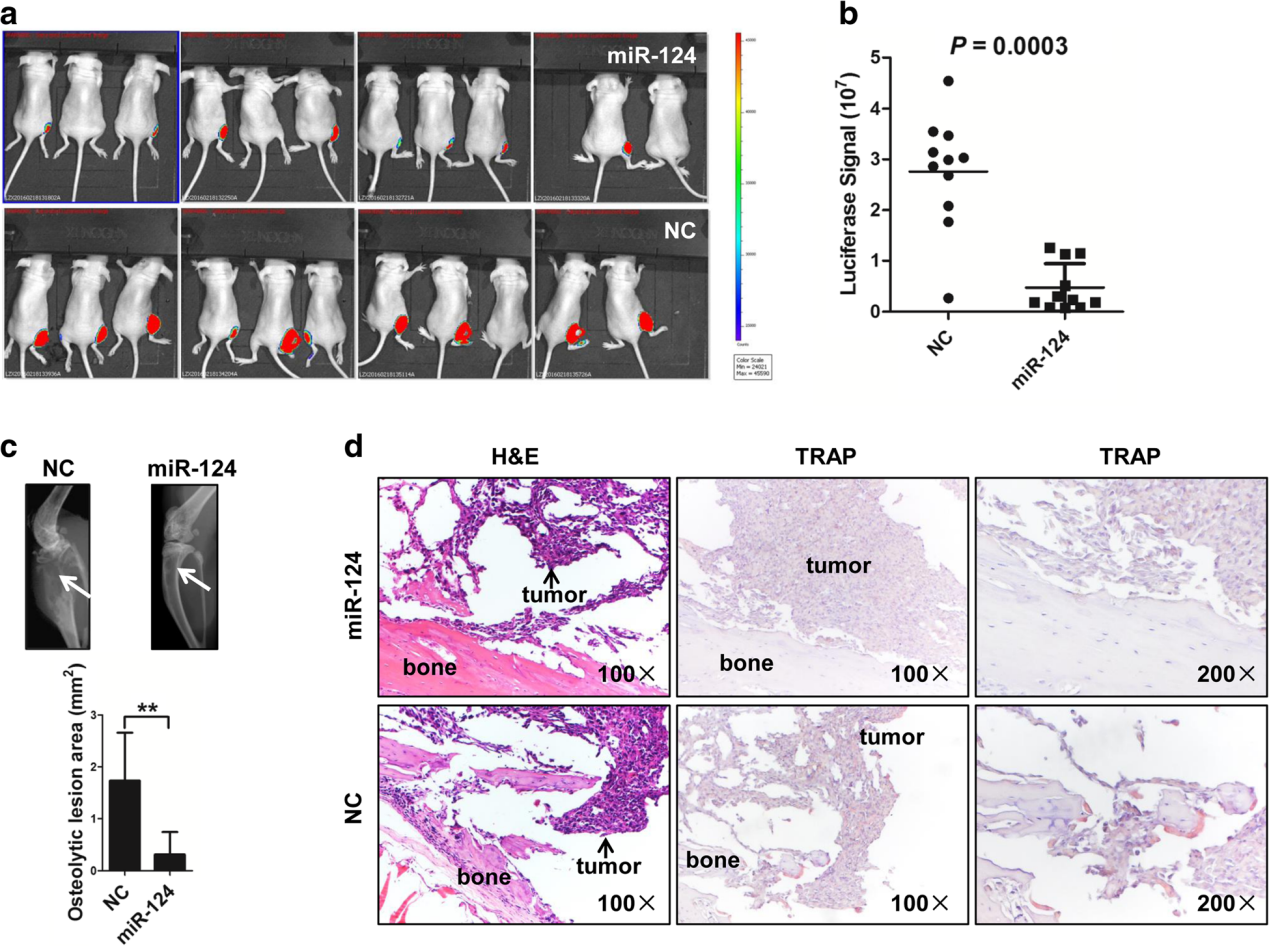
miR-124 reinforcement inhibits survival of breast cancer cells in bone microenvironment. **a** Images of luciferase signal in tibias of nude mice transplanted with luciferase-labeled MDA-MB-231 cells overexpressing miR-124 or negative control (NC). **b** miR-124 overexpression reduced the quantification of bone lesions detected by bioluminescence. Horizontal line indicates median value. *P* = 0.0003 by Mann–Whitney U test. **c** Effects of miR-124 on breast cancer-induced osteolytic lesions in mice as shown by X-ray imaging. Arrows indicate osteolytic lesions (Top). Statistical analysis showed reduced osteolytic lesion area in miR-124 group (Bottom). **d** Effects of miR-124 on the number and activity of osteoclasts in the tumor-bone interface as detected by TRAP staining (red signal). Mouse tibias were collected from each group and sectioned for H&E staining (left) at an original magnification of ×100 and TRAP staining (right) at an original magnification of ×100 and ×200

To investigate the effect of miR-124 on survival of breast cancer cell in the bone microenvironment, luciferase-labeled MDA-MB-231 cells infected with lentivirus expressing miR-124 or control lentivirus (negative control, NC) (Additional file 1: Figure S1) were transplanted into Balb/c nude mice via the intratibia route. Luciferase signals detected in tibias using ex vivo imaging 4 weeks after injection were significantly lower in mice expressing miR-124 than in mice from control group (Fig. 2a, b). Moreover, X-ray analysis indicated that cancer cell-induced osteolysis was repressed in the miR-124 group (Fig. 2c). TRAP staining showed that both the number and activity of osteoclasts were markedly reduced at the boundary in the miR-124 group (Fig. 2d).

### miR-124 inhibits and prevents bone metastasis of breast cancer cell in mice

**Fig. 3:**
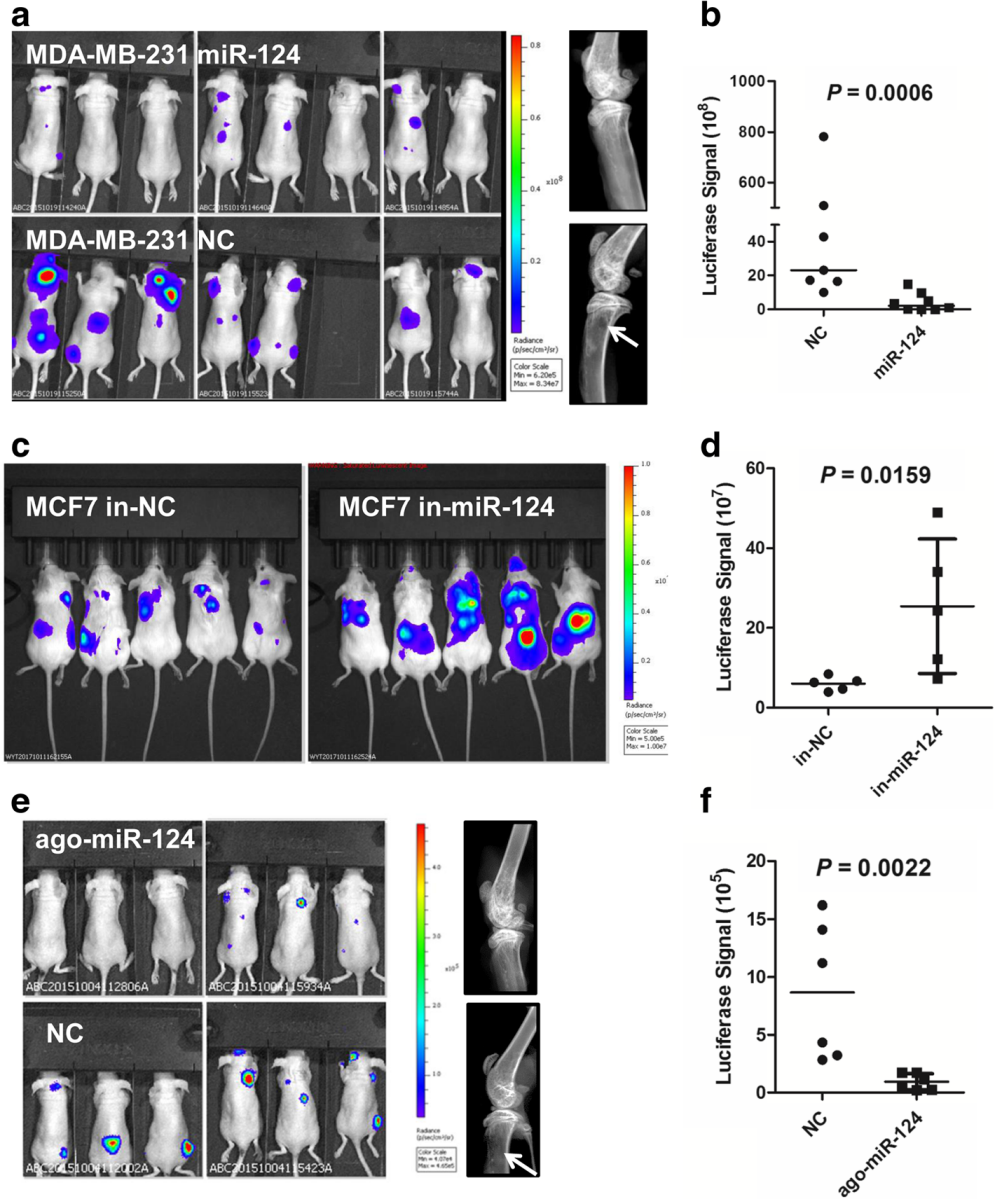
The effect of miR-124 on bone metastasis from breast cancer cells in mice. **a** Images of luciferase signal in nude mice inoculated with luciferase-labeled MDA-MB-231 cells stably expressing miR-124 or NC into the left ventricle (Left). Representative X-ray imagings of tibias from mice are shown on the right. The arrow indicates osteolytic lesion. **b** Statistical analysis showed reduced luciferase signal in nude mice inoculated with luciferase-labeled MDA-MB-231 cells stably expressing miR-124. Horizontal line indicates median value. *P* = 0.0006 by Mann–Whitney U test. **c** Images of luciferase signal in nude mice inoculated with luciferase-labeled MCF7 cells stably expressing miR-124 inhibitor (in-miR-124) or NC (in-NC) into the left ventricle. **d** Statistical analysis showed a significant increase of luciferase signal in nude mice inoculated with luciferase-labeled MCF7 cells stably expressing miR-124 inhibitor. Horizontal line indicates median value. *P* = 0.0159 by Mann–Whitney U test. **e** Images of luciferase signal in nude mice inoculated with MDA-MB-231 cells and then injected with ago-miR-124 or NC (Left). Representative X-ray imagings of tibias from mice are shown on the right. The arrow indicates osteolytic lesion. **f** Statistical analysis showed significantly lower luciferase signal in nude mice inoculated with luciferase-labeled MDA-MB-231 cells and then injected with ago-miR-124 than in mice injected with NC. Horizontal line indicates median value. *P* = 0.0022 by Mann–Whitney U test

We further evaluated the effect of miR-124 on bone metastasis of breast cancer cells in vivo by inoculating luciferase-labeled MDA-MB-231 cells stably expressing miR-124 or NC, as well as luciferase-labeled MCF7 cells stably expressing miR-124 inhibitor or NC, into the left ventricles of Balb/c nude mice. In mice administered MDA-MB-231 cells stably expressing NC, 25% (2 of 8) showed bone metastasis after 1 week by ex vivo imaging, including metastases to the skull, spine and tibia, and all of the subjects exhibited bone metastases 3 weeks after inoculation, with one mice died in the third week (Additional file 1: Figure S2a, Figure S3a). However, no signal was observed until day 21 in mice injected with cells stably expressing miR-124, and significantly lower signals were identified in 37.5% (3 of 8) of mice 5 weeks after inoculation (Additional file 1: Figure S2a, Figure S3a, b). X-ray analysis indicated inhibition of cancer cell-induced osteolysis in the miR-124 group (Fig. 3a), and the following hematoxylin-eosin (H&E) staining confirmed bone lesions in the control group mice (Additional file 1: Figure S2b). Moreover, luciferase signals were significantly higher in mice administered MCF7 cells stably expressing miR-124 inhibitor than those in mice administered cells stably expressing NC (Fig. 3c, d). Metastases to the bones including ribs, spine and tibias, as detected by luciferase signals, were also significantly increased in mice administered MCF7 cells stably expressing miR-124 inhibitor than those in mice from NC group (Additional file 1: Figure S3a, b). In addition, metastases to the lungs, as detected by luciferase signals and H&E staining, were identified in 60% (3/5) of the mice of the miR-124 inhibitor group, while no lung metastasis was observed in the mice of the NC group (Additional file 1: Figure S3c, d). The luciferase signals in the hearts were emitted by residual cells after ventricle inoculation. To explore whether miR-124 might be applied to prevent bone metastasis, luciferase-labeled MDA-MB-231 cells were inoculated into the left ventricle of nude mice followed by injections of ago-miR-124 or NC via the tail vein. Luciferase signals were significantly lower in the ago-miR-124 group compared to the control group (Fig. 3e, f). Strikingly, only 50% (3 of 6) of mice in the agomiR-124 group developed tiny bone metastases, whereas all of the mice in the control group developed obvious bone metastases (Fig. 3e, f). X-ray analysis and H&E staining showed a further reduction of tumor-induced osteolysis in the ago-miR-124 group (Fig. 3e, Additional file 1: Figure S4).

### miR-124 inhibits the survival and differentiation of osteoclast progenitor cells in vitro

**Fig. 4:**
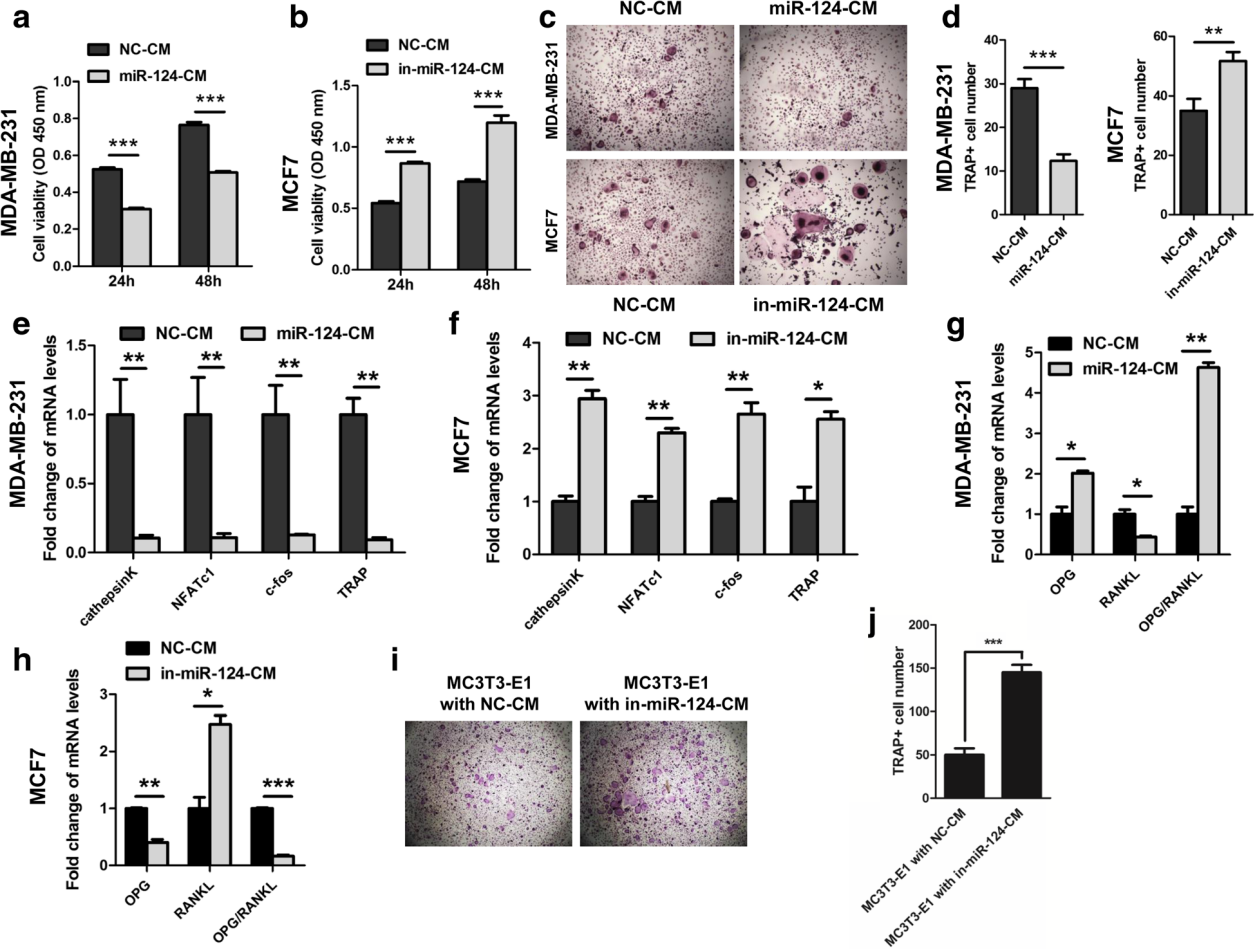
miR-124 inhibits osteoclastogenesis by regulating osteoclast and osteoblast in vitro. **a** BMMs were cultured in α-MEM containing conditioned medium from MDA-MB-231 cells transfected with miR-124 mimic (miR-124-CM) or NC (NC-CM). Surviving cells were counted at 24 h and 48 h. **b** Cultured with α-MEM containing conditioned medium from MCF7 cells transfected with miR-124 inhibitor (in-miR-124-CM) or inhibitor NC (NC-CM) reduced the cell viability of BMMS. **c** and **d** The effects of miR-124-CM from MDA-MB-231 cells and in-miR-124-CM from MCF7 cells on RANKL-induced mouse BMM differentiation as detected by TRAP staining. Representative images of TRAP staining (original magnification, ×40) were showed in (c) and statistical analyses were showed in (d). Columns show the means of performed experiments in triplicate; bars show SD. **e** and **f** Real-time RT-PCR analysis of Cathepsin K, NFATc1, c-fos and TRAP expression in RAW264.7 cells treated with miR-124-CM or NC-CM from MDA-MB-231 cells (e) as well as in RAW264.7 cells treated with in-miR-124-CM or NC-CM from MCF7 cells (f). Gene expression was normalized against β-actin. **g** and **h** The effect of miR-124-CM from MDA-MB-231 cells (g) and in-miR-124-CM from MCF7 cells (h) on OPG and RANKL expression in MC3T3-E1 cells. **i** and **j** MC3T3-E1 cells were firstly cultured with conditioned medium from MCF7 cells transfected with miR-124 inhibitor or NC, and then conditioned medium of MC3T3-E1 from both groups were collected (MC3T3-E1 with in-miR-124-CM or MC3T3-E1 with NC-CM). BMMs were cultured in α-MEM containing conditioned medium from MC3T3-E1 with in-miR-124-CM or MC3T3-E1 with NC-CM. Representative images of TRAP staining (original magnification, ×40) were showed in (i) and statistical analyses were showed in (j). * P < 0.05, ** *P* < 0.01 and *** *P* < 0.001 by two-tailed Student’s t test. Experiments were performed in triplicate and data are shown as mean ± SD

The role of miR-124 in the metastasis of breast cancer cells to bone is largely unknown. The reduced osteolysis observed in mice injected with miR-124 expressing cells raises the possibility that cancer cell-derived miR-124 might inhibit osteoclastogenesis by regulating osteoclast and osteoblast activity. To verify this hypothesis, we selected BMMs with M-CSF stimulation as an in vitro osteoclast differentiation model. We first cultured BMMs, using conditioned media from MDA-MB-231 (with low expression of miR-124) transfected with miR-124 mimic (miR-124-CM) or NC (NC-CM) as well as conditioned media from MCF7 (with high expression of miR-124) transfected with miR-124 inhibitor (in-miR-124-CM) or NC inhibitor (NC-CM). Cultured with miR-124-CM significantly decreased, while cultured with in-miR-124-CM increased the number of BMMs (Fig. 4a, b). Second, after treatment with M-CSF and RANKL, BMMs cultured in miR-124-CM showed fewer cells differentiated into TRAP-positive multinucleated osteoclasts than BMMs cultured in NC-CM (Fig. 4c, d), and miR-124-CM also reduced the formation of actin-ring structures (Additional file 1: Figure S5). In contrast, in-miR-124-CM significantly enhanced the differentiation of BMMs as detected by TRAP staining (Fig. 4c, d). Cathepsin K, NFATc1, c-fos and TRAP are important transcription factors involved in osteoclastogenesis [34]. Cultured with miR-124-CM markedly promoted, whereas cultured with in-miR-124-CM suppressed the expression of these transcription factors in osteoclast cell line RAW264.7 (Fig. 4e, f). RANKL is the most prominent cytokine inducer of osteoclastogenesis [7], and RANKL activity is balanced in normal bone homeostasis by osteoprotegerin (OPG), a decoy RANKL receptor secreted by osteoblasts [7]. Therefore, in the bone microenvironment, tumor cell-induced osteoclastogenesis involves a reduction of the OPG/RANKL ratio in osteoblasts. In this study, we cultured osteoblast-like cells MC3T3-E1 with miR-124-CM and NC-CM or in-miR-124-CM and NC-CM, and real-time RT-PCR showed increased OPG and decreased RANKL expression as well as an increased OPG/RANKL ratio in MC3T3-E1 cultured with miR-124-CM (Fig. 4g). miR-124-CM also inhibited MMP13 expression, which is secreted from osteoblasts and stimulates osteoclast differentiation and activation [35], in MC3T3-E1 (Additional file 1: Figure S6). In contrast, in-miR-124-CM reduced the expression of OPG, enhanced the expression of RANKL, thus decreasing the ration of OPG/RANKL (Fig. 4h). We further collected the conditioned media from MC3T3-E1 cells cultured with in-miR-124-CM and NC-CM from MCF7 cells. The results showed that the conditioned media from MC3T3-E1 cells cultured with in-miR-124-CM significantly enhanced the differentiation of BMMs as detected by TRAP staining (Fig. 4i, j). In addition, the cancer cell-derived osteoclast-activating factors MCSF, IL-6, IL-8, RANKL, MMP2, MMP13, RUNX2 and PTHrP [6] are significantly down-regulated in MDA-MB-231 cells transfected with miR-124 mimic (Additional file 1: Figure S7a) and up-regulated in MCF7 cells transfected with miR-124 inhibitor (Additional file 1: Figure S7b). Collectively, these results suggest that miR-124 inhibits osteoclastogenesis both directly and indirectly by acting on osteoclasts.

### IL-11 is directly down-regulated by miR-124

**Fig. 5:**
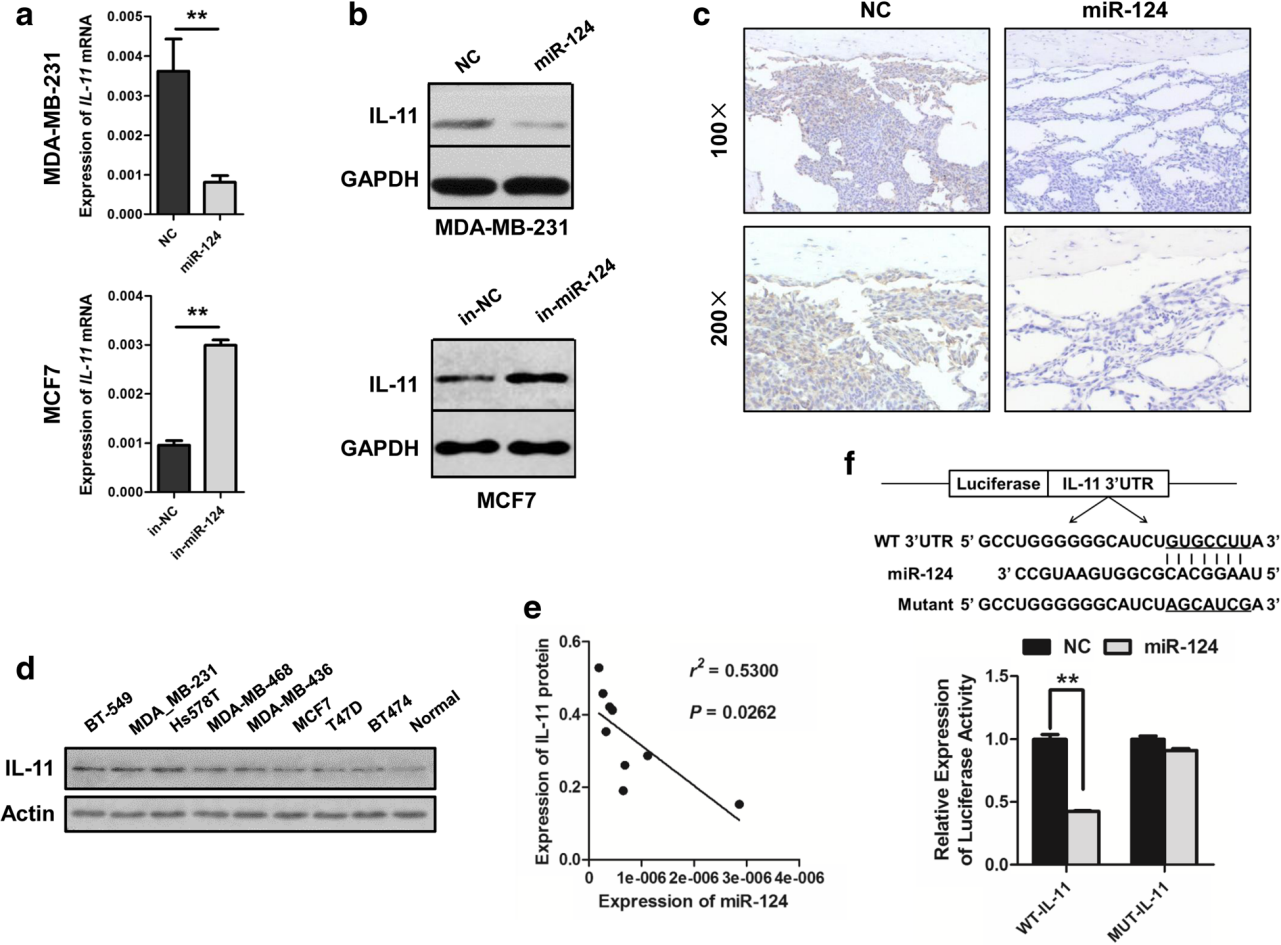
Identification of IL-11 as a bona fide target of miR-124. **a** and **b** Real-time RT-PCR analysis of *IL-11* mRNA expression (a) or western blot analysis of IL-11 protein expression (b) in MDA-MB-231 cells transfected with miR-124 mimic (miR-124) or negative control (NC) (top) and in MCF7 cells transfected with miR-124 inhibitor (in-miR-124) or negative control (NC) (bottom). **c** IHC staining analysis of IL-11 expression in the tibia from mice injected with MDA-MB-231 cells stably expressing miR-124 (miR-124) or NC. **d** IL-11 protein expression was determined by Western blot in normal human breast tissue (Normal) and eight kinds of human breast cancer cell lines. **e** Expression of IL-11, as determined by semi-quantitative analysis using BandScan, in normal human breast tissue and human breast cancer cell lines was negatively related to the level of miR-124 as detected by Real-time RT-PCR. r^2^ = 0.5300, *P* = 0.0262 by Spearman correlation analysis. **f** Reporter assay identified IL-11 as a direct target of miR-124. Sketch of the construction of wild type (WT) or mutant psicheck2-IL-11 3’UTR vectors. The seed sequence and mutant binding sites are underlined (top). Luciferase reporter plasmid carrying *IL-11* 3’UTR (WT-IL-11) or *IL-11* 3’UTR with point mutation in miR-124 target sequence (MUT-IL-11) was cotransfected into HEK293T cells with miR-124 mimic or NC (bottom)

To investigate the underlying molecular mechanisms by which miR-124 exerts its anti-bone-metastasis effect, we screened for putative miR-124 targets by performing an in silico complementarity search using TARGETSCAN-VERT (www.targetscan.org/), MICRORNA.ORG (www.microrna.org/), MIRDB (http://mirdb.org/) and TARBASE (http://diana.imis.athena-innovation.gr/) on key regulators of osteoclastogenesis. These screening algorithms overlapped on IL-11, indicating that IL-11 is a potential downstream miR-124 target. Additional assays showed that IL-11 mRNA and protein levels were decreased in MDA-MB-231 by ectopic miR-124 expression and increased in MCF7 cells transfected with miR-124 inhibitor (Fig. 5a, b). Furthermore, IL-11 level was reduced in the tibia of mice injected with MDA-MB-231 cells stably expressing miR-124 (Fig. 5c). Western blotting indicated that IL-11 protein expression was substantially decreased in non-invasive cell lines (MCF7, T47D, BT-474) compared with invasive cell lines (BT-549, MDA-MB-231, Hs578T, MDA-MB-468 and MDA-MB-436) and was lowest in normal human breast tissue (Fig. 5d), which is the inverse of miR-124 expression (Fig. 1a). Indeed, an association study showed a negative correlation between miR-124 and IL-11 protein expression (Fig. 5e). Moreover, IL-11 expression was enhanced in MDA-MB-231-B compared to MDA-MB-231-P (Additional file 1: Figure S8), and a reporter assay revealed that miR-124 overexpression reduced luciferase activity from the wild-type (WT) *IL-11* 3′ untranslated region (UTR) by 57.4% (Fig. 5f). Point mutation of the target sequence in the *IL-11* 3′ UTR diminished the miR-124 effect, indicating that IL-11 is a direct downstream target of miR-124 (Fig. 5f).

### miR-124 regulates osteoclastogenesis and inhibits bone metastasis by inhibiting IL-11

**Fig. 6:**
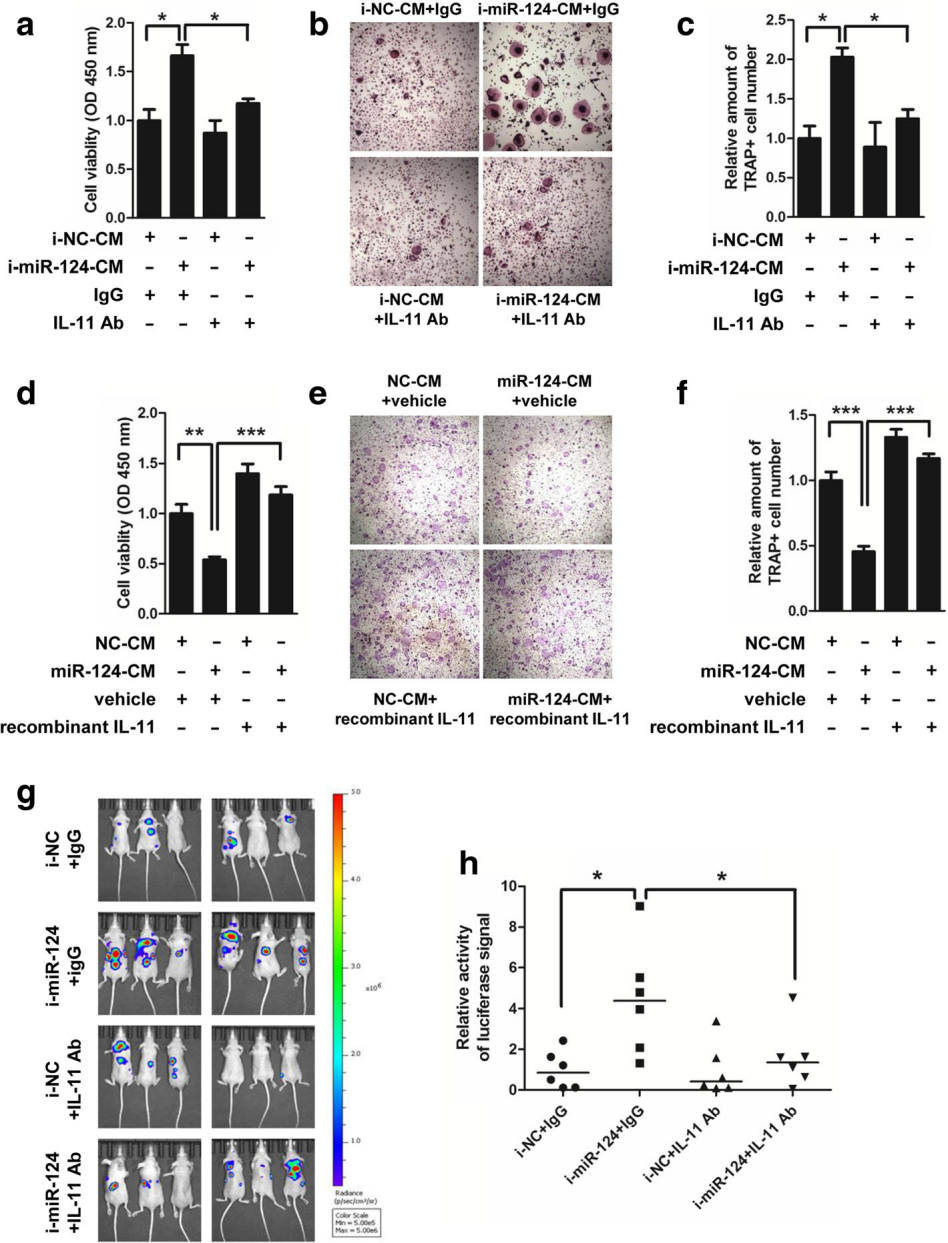
IL-11 partially mediates the inhibition breast cancer bone metastasis by miR-124 both in vitro and in vivo. **a-c** IL-11 neutralizing antibody treatment (5 μg/ml) efficiently reversed the promoting effects of conditioned medium from MDA-MB-231 cells transfected with miR-124 inhibitor (i-miR-124-CM) on the proliferation (a) and differentiation of BMMs as determined by TRAP staining (b and c). Representative images of TRAP-positive multinucleated osteoclasts are shown in b and statistical analysis is shown in c. **d-f** Recombinant human IL-11 treatment (10 ng/ml) partially reversed the suppressive effects of conditioned medium from MDA-MB-231 cells transfected with miR-124 (miR-124-CM) on the proliferation (d) and differentiation of BMMs as determined by TRAP staining (e and f). * P < 0.05, ** *P* < 0.01 and *** *P* < 0.001 by two-tailed Student’s t test. Experiments were performed in triplicate and data are shown as mean ± SD. **g** MCF7 cells stably expressing miR-124 inhibitor (i-miR-124) or NC (i-NC) were firstly inoculated into the left ventricle of nude mice, and then 5 μg IL-11 neutralizing antibody (IL-11 Ab) or the control IgG (IgG) were injected into the tail veins of the mice. Luciferase signals were measured with an ex vivo imaging system. **h** Statistical analysis showed IL-11 neutralizing antibody partially reverses the promoting effect of miR-124 inhibitor on the metastasis of MCF7 cells. Horizontal line indicates median value. * P < 0.05 by Mann–Whitney U test

To verify that IL-11 is not only a direct target but also a functional effector of miR-124 when regulating osteoclastogenesis, we performed a series of rescue assays using the commercial IL-11 neutralizing antibody and recombinant human IL-11 both in vitro and in vivo. First, BMMs treated with M-CSF and RANKL and cultured in conditioned medium from MDA-MB-231 cells transfected with miR-124 inhibitor (i-miR-124-CM) displayed higher proliferation and more differentiation to TRAP-positive multinucleated osteoclasts than those cultured in the control group, whereas IL-11 neutralizing antibody treatment efficiently reversed the effect of i-miR-124-CM on proliferation and differentiation of osteoclast progenitor (Fig. 6a-c). Consistently, BMMs cultured in conditioned medium from MDA-MB-231 cells transfected with miR-124 mimic (miR-124-CM) displayed lower proliferation and less differentiation to TRAP-positive multinucleated osteoclasts than those cultured in the control group, whereas recombinant IL-11 treatment substantially reversed these effects (Fig. 6d-f). Next, IL-11 neutralizing antibody reversed i-miR-124-CM mediated increase of Cathepsin K, NFATc1, c-fos and TRAP expression in RAW264.7 cells, while recombinant IL-11 reversed miR-124-CM mediated decrease of these genes levels in RAW264.7 cells (Additional file 1: Figure S9a, b). Thirdly, the suppressive effect of i-miR-124-CM on the ratio of OPG to RANKL was substantially inhibited by IL-11 neutralizing antibody treatment, while the promoting effect of miR-124-CM on the ratio of OPG to RANKL was partially reversed by recombinant IL-11 (Additional file 1: Figure S9c, d). Importantly, in vivo assay showed that IL-11 neutralizing antibody could partially reverse the promoting effect of miR-124 inhibitor on the bone metastasis and lung metastasis of breast cancer cells in mice (Fig. 6g, h, Additional file 1: Figure S10, Figure S11). Together, these findings demonstrate that IL-11 down-regulation contributes to the function of miR-124 in bone metastasis of breast cancer cells both in vitro and in vivo.

### Perturbation of the miR-124/IL-11 regulatory axis is associated with clinicopathological characteristics and the survival of breast cancer patients with bone metastasis

**Fig. 7:**
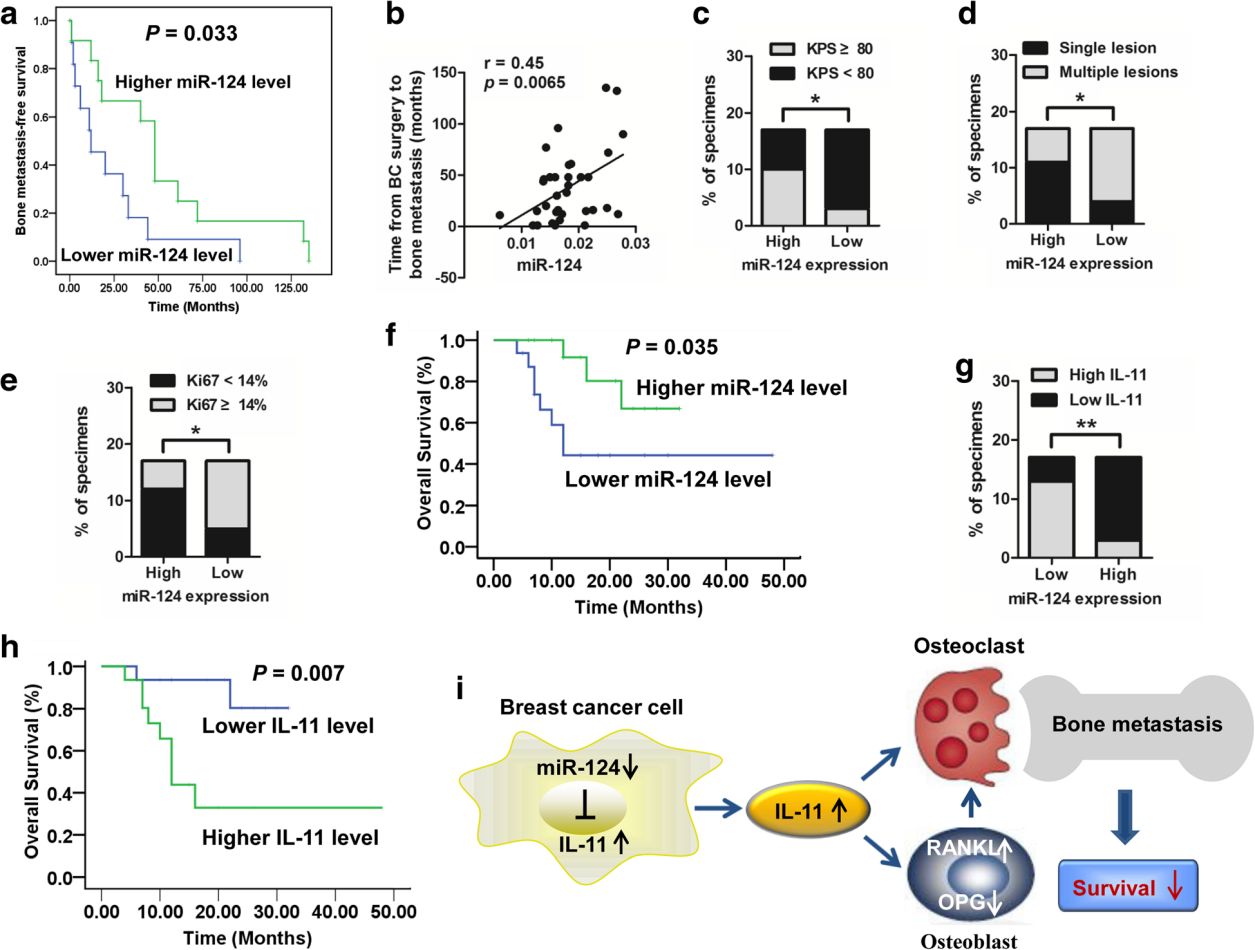
The miR-124/IL-11 axis is perturbed in breast cancer patients with bone metastasis. **a** Kaplan-Meier analysis for bone metastasis-free survival of 79 patients who received breast cancer surgery and a follow-up of the development of bone metastasis. The median value of miR-124 expression in primary breast cancer tissues as detected by ISH from all 79 samples was chosen as the cut-off point. *P* = 0.033 by log rank test. **b** A correlation analysis was performed between miR-124 expression of bone metastasis tissues from 34 patients that received bone metastasis resection and the time from primary breast cancer surgery to bone metastasis development. *r* = 0.45, *p* = 0.0065 by Spearman correlation analysis. **c-e** Percentages of specimens with low or high miR-124 expression were relative to percentages of specimens with KPS ≥ 80 or KPS < 80 (c), and specimens with single bone lesion or multiple bone lesions (d) as well as specimens with Ki67 < 14% or Ki67 ≥ 14% (e). * *P* < 0.05 by Fisher’s exact test. **f** Kaplan-Meier analysis for the overall survival of 34 patients with bone metastasis. The median value of all 34 samples was chosen as the cut-off point. *P* = 0.035 by log rank test. **g** Percentages of specimens with low or high miR-124 expression relative to the levels of IL-11 protein. ** *P* < 0.01 by Fisher’s exact test. **h** Kaplan-Meier analysis was used to compare the overall survival of patients with higher expression of IL-11 and lower expression of IL-11. The median value of IL-11 in all 34 samples was chosen as the cut-off point. *P* = 0.007 by log rank test. **i** A schematic representation of the miR-124/IL-11 regulatory axis in the interaction between primary breast cancer and bone microenvironment

To further validate the clinical relevance of miR-124 in bone metastasis of breast cancer patients, we first performed a Kaplan-Meier analysis in patients who received breast cancer surgery and then a follow-up of the development of bone metastasis. The results showed lower miR-124 levels in primary breast cancer tissues were correlated with shorter bone metastasis-free survival (Fig. 7a). Consistently, correlation analysis revealed that miR-124 levels in metastatic bone tissues were positively correlated with the time from the primary breast cancer surgery to the occurrence of bone metastasis, and the progression time in patients with high miR-124 expression was longer than in patients with low miR-124 expression (Fig. 7b, Additional file 1: Figure S12), indicating that patients with lower miR-124 expression might progress to bone metastasis earlier. Of interest, clinicopathological analysis demonstrated that miR-124 down-regulation in human metastatic bone tissues was significantly correlated with aggressive clinicopathological characteristics, including poorer quality of life as evaluated by the Karnofsky performance score (KPS) (*P* = 0.0324), a larger number of metastatic bone lesions (*P* = 0.0366) and a higher proliferation potential as assessed by Ki67 staining (*P* = 0.0381) (Fig. 7c-e and Additional file 1: Table S1). Importantly, Kaplan-Meier analysis revealed that lower miR-124 levels in metastatic bone tissues were correlated with shorter overall survival of patients (Fig. 7f). As a functional downstream miR-124 target, IL-11 protein expression detected by IHC analysis was enhanced in the primary breast cancer compared to paired non-tumor tissues and was further elevated in metastatic bone tissues (Additional file 1: Figure S13) as opposed to the miR-124 expression in human tissues (Fig. 1d, e). Indeed, IL-11 expression was negatively correlated with miR-124 expression in paired primary breast cancer tissues and adjacent non-tumorous mammary tissues as well as bone metastases from the breast cancer (Fig. 7g). Furthermore, IL-11 expression was negatively correlated to the time from breast cancer surgery to bone metastasis development (Additional file 1: Figure S14a). In contrast to miR-124, the progression time in patients with high IL-11 expression was shorter than in those with low IL-11 level (Additional file 1: Figure S14b), indicating that patients with higher IL-11 expression might develop bone metastasis earlier. Clinicopathological analysis also indicated that IL-11 up-regulation in human metastatic bone tissues was significantly related to aggressive clinicopathological characteristics, including a larger number of metastatic bone lesions (*P* = 0.0366) and a higher proliferation potential as assessed by Ki67 staining (*P* = 0.0004) (Additional file 1: Figure S15, Table S2). Intriguingly, in contrast to the prognostic significance of miR-124, higher IL-11 expression in metastatic bone tissues was correlated with shorter overall survival for patients with bone metastasis (Fig. 7h). Overall, these findings suggest that dysregulation of the miR-124/IL-11 axis affects tumor-stromal interactions during the bone metastasis of breast cancer, thus exerting a negative influence on clinicopathological characteristics and the survival of breast cancer patients with bone metastasis (Fig. 7i).

## Discussion

Previous studies have demonstrated that miR-124 inhibits the proliferation, epithelial-mesenchymal transition (EMT), migration, invasion and angiogenesis of breast cancer cells [23–28]. However, the function of miR-124 in bone metastasis remains elusive. Moreover, it has been reported that the introduction of the miR-124 precursor into BMMs dramatically reduced RANKL-dependent osteoclast differentiation, whereas an miR-124 inhibitor potently enhanced osteoclastogenesis [36], indicating the crucial role of miR-124 in bone homeostasis, but it is unknown whether tumor cell-derived miR-124 contributes to the interaction between tumor cells and the bone microenvironment. This study revealed that miR-124 expression was reduced in breast cancer cell lines, especially those with high invasive potential. A substantial miR-124 decrease was also observed in a MDA-MB-231 derivative cell line with strong bone-metastatic property compared to the parental cell line. Moreover, the in vivo assays demonstrated that miR-124 suppressed breast cancer cell survival in the bone microenvironment and cancer cell colonization of bone. Mechanistically, tumor cell-derived miR-124 inhibited osteoclastogenesis by acting on osteoclasts and osteoblasts, whereas miR-124 inhibition exerted the opposite effects. These data not only confirm the well-recognized anti-neoplastic properties of miR-124 in breast cancer but also describe an unprecedented role of miR-124 in the progression of breast cancer to bone.

To date, bone modifying agents, including bisphosphonates and denosumab, can prevent and treat SREs by inhibiting osteoclast formation and bone degradation and have been viewed as an indispensable therapy for breast cancer patients with bone metastasis [37]. Moreover, meta-analyses have verified that adjuvant bisphosphonates reduce the rate of breast cancer recurrence in bone and improve breast cancer survival, but the benefit is only observed in women who were postmenopausal at the onset of treatment [38]. Recently, a European Panel released consensus guidance for the clinical practice of using adjuvant bisphosphonates to treat early breast cancer; however, considering both toxicity and adherence, these applications should be confined to select patients who are suitable for adjuvant bisphosphonate treatment to prevent bone metastases [39]. In this study, miR-124 expression was reduced in primary breast cancer tissues and further depressed in metastatic bone lesions, suggesting that miR-124 down-regulation might be a pivotal event in bone metastasis development. As expected, fewer metastatic bone lesions were found in mice with breast cancer cells stably expressing miR-124 injected into the left cardiac ventricle than in the control group, while miR-124 inhibitor exerted the opposite effects. Consistently, systemic delivery of synthetic miRNA-124 inhibited the formation of bone metastases in mice. These findings indicate that miR-124 reintroduction might be a promising adjuvant therapy to prevent bone metastasis in patients with early breast cancer. Furthermore, as endogenous non-coding double-strand RNAs, miRNAs are characterized by tissue specificity and molecular targeting. Hence, externally engineering miR-124 expression might be safer than traditional bone modifying agents.

IL-11, which is produced by several cell types in the tumor-stromal microenvironment, such as tumor cells, fibroblasts and osteoblasts [10], is a cytokine that promotes osteolysis by stimulating osteoclast formation [40]. It was first shown to enhance osteoclast development through a mechanism requiring osteoblasts, because it has the dual function of inhibiting OPG production and stimulating RANKL production in osteoblasts [41, 42]. A later study verified that IL-11 promotes osteoclast formation independent of RANKL [43]. Moreover, a recent study indicated that breast cancer cell-derived IL-11 augments osteoclastogenesis by stimulating the development and/or survival of osteoclast progenitor cells [10]. Therefore, IL-11 dysregulation in breast cancer cells contributes to osteolysis development in multiple ways, although the upstream regulator of IL-11 in tumor cells has yet to be discovered. This study demonstrated that miR-124 negatively regulates IL-11 expression in vitro and in vivo. There was a negative correlation between miR-124 levels and IL-11 expression both in cell lines and in human metastatic bone tissues. Reporter assays further confirmed that IL-11 was a bona-fide miR-124 target. More importantly, in vitro and in vivo assays demonstrated the active role of IL-11 down-regulation in the breast cancer-derived miR-124-mediated suppression of osteoclastogenesis and bone metastasis. These findings not only reveal a novel role for miR-124 in regulating IL-11 but also confirm the comprehensive effects of IL-11 in the promotion of osteoclastogenesis.

Decreased miR-124 expression has been reported to be an unfavorable independent prognostic factor for patients with breast cancer [29], whereas high IL-11 expression correlates with high histological grade and poor survival in breast cancer [44, 45]. Nevertheless, the specific clinical significance of miR-124 and IL-11 in bone metastasis was unclear. Our data in this study demonstrate that miR-124 expression in primary breast cancer is correlated with bone metastasis-free survival of patients. Furthermore, miR-124 expression in metastatic bone tissues is positively correlated and IL-11 is negatively correlated with the time from primary breast cancer surgery to the development of bone metastasis. Specifically, patients with lower miR-124 expression or higher IL-11 expression in metastatic bone tissues might experience shorter progression time, and vice versa. Quality of life is of important clinical significance for patients with late-stage breast cancer, and the number of lytic bone lesions suggests the bone-metastatic potential of primary breast cancer. Ki67, a marker associated with cell proliferation, can distinguish luminal B breast cancer, which is associated with poor recurrence-free and disease-specific survival, from luminal A tumors at the cut point of 14% [46]. Our correlation analysis further highlights the negative relationship of miR-124 levels with aggressive clinicopathological characteristics including preoperative KPS, number of metastatic bone lesions and Ki67 index as detected by IHC, and the positive association of IL-11 expression with number of metastatic bone lesions as well as Ki67 index. More importantly, survival analysis showed that a lower miR-124 level and higher IL-11 expression in metastatic bone tissues was correlated with shorter overall survival of patients with bone metastasis. Collectively, these results suggest that the functional loss of miR-124 might result in enhanced IL-11 expression, which promotes the development of osteolytic lesions and eventually favors tumor progression.

## Conclusion

Our data reveal an miRNA-dependent regulatory axis that links the well-known tumor suppressor miR-124 to IL-11-induced osteolysis, which when disrupted in breast cancer might be associated with bone metastasis development and subsequently a poor prognosis. Consequently, miR-124 and IL-11 might be new therapeutic targets and prognostic markers for breast cancer patients at early stage and at advanced stage with bone metastasis.

## Additional file

Additional file 1: Supplemental Information. (DOCX 7445 kb)

## Abbreviations

3′UTR: 3’untranslated regions
BMMs: Bone marrow monocytes
EMT: epithelial-mesenchymal transition
FACS: Fluorescence-activated cell sorting
FBS: Fetal bovine serum
GFP: Green fluorescent protein
H&E: Hematoxylin-eosin
IHC: Immunohistochemical
IL-11: Interleukin-11
IL-6: Interleukin-6
ISH: In situ hybridization
KPS: Karnofsky performance score
microRNAs: miRNAs
miR-124: microRNA-124
NC: Negative control
OPG: Osteoprotegerin
PBS: Phosphate-buffered saline
RANKL: The receptor activator of nuclear factor-κB ligand
SREs: Skeletal-related events
TRAP: Tartrate resistant acid phosphatase
WT: Wild type
