# Correction to: microRNA-124 inhibits bone metastasis of breast cancer by repressing Interleukin-11

**DOI:** 10.1186/s12943-020-01226-1

**Published:** 2020-06-27

**Authors:** Wei-Luo Cai, Wen-Ding Huang, Bo Li, Tian-Rui Chen, Zhen-Xi Li, Cheng-Long Zhao, Heng-Yu Li, Yan-Mei Wu, Wang-Jun Yan, Jian-Ru Xiao

**Affiliations:** 1 Department of Musculoskeletal Tumor, Fudan University Shanghai Cancer Center; Department of Oncology, Shanghai Medical College, Fudan University, Shanghai, 200032 China; 2 Spine Tumor Center, Changzheng Hospital, Second Military Medical University, Shanghai, 200003 China; 3 Department of Breast and Thyroid Surgery, General Surgery, Changhai Hospital, Second Military Medical University, Shanghai, 200433 China

**Correction to: Molecular Cancer (2018) 17:9**

**https://doi.org/10.1186/s12943-017-0746-0**

After publication of the article [1], the authors reported errors of inter-duplication in Fig. 4c. As shown in the original Fig. 4c, the “TRAP staining” images of “MDA-MB-231 NC-CM” and “MDA-MB-231 miR-124-CM” were shown identical to “i-miR-124-CM + IL-11 Ab” and “i-NC-CM+ IL-11 Ab” group in Fig. 6b. The authors have confirmed that the “TRAP staining” images of “i-miR-124-CM + IL-11 Ab” and “i-NC-CM+ IL-11 Ab” in Fig. 6b were mistakenly presented in the original Fig. 4c. This mistake was caused by unintentionally covering the correct image during figure preparation, which is reflected by the original images. The updated figure (Fig. 4c) is included in this correction. The correction does not affect the findings or conclusions of the article. The authors apologize for any inconvenience that the inaccuracy may have caused.

The correct figures are updated below.


