# Supplementary material for: microRNA-124 inhibits bone metastasis of breast cancer by repressing Interleukin-11
Source: Mol Cancer. 2018 Jan 17;17:9. doi: 10.1186/s12943-017-0746-0 (PMC5773190; doi:10.1186/s12943-017-0746-0)
Supplement: Additional file 1: — Supplemental Information. (DOCX 7445 kb) [file 12943_2017_746_MOESM1_ESM.docx]

**Supplementary Materials**

**Supplementary Methods**

**Cell culture**

Human breast cancer cell lines (BT-549, MDA-MB-231, Hs578T, MDA-MB-468, MDA-MB-436, MCF7, T47D and BT-474) as well as RAW264.7 and MC3T3-E1 were obtained from ATCC. The strongly bone-metastatic MDA-MB-231-derived subline, MDA-MB-231-luc-D3H2LN, was purchased from Xenogen Corporation (Alameda, CA, USA). BT-549, MDA-MB-231, Hs578t, MDA-MB-468, MDA-MB-436, BT-474, RAW264.7, MC3T3-E1 and MDA-MB-231-luc-D3H2LN were maintained in DMEM (GIBCO / Thermo Fisher, Waltham, MA, USA) supplemented with 10% fetal bovine serum (FBS) (HyClone / GE Life Sciences, Logan, UT, USA) in an incubator (37°C, 5% CO2). MCF7 and T47D were maintained in RPMI1640 (GIBCO / Thermo Fisher) supplemented with 10% FBS. Bone marrow-derived macrophages (BMMs) were isolated from C57/BL6 mice as described previously^[1]^ and cultured in DMEM supplemented with 10% FBS. All of the cell lines were tested using MycoAlert Mycoplasma Detection Kit (Lonza, Switzerland) according to the manufacturer’s instructions and were free of mycoplasma contamination.

**Clinical tissues**

Primary breast cancer tissues and adjacent non-tumorous mammary tissues were obtained from patients who received breast cancer surgery and were histopathologically verified at Changhai hospital. Bone metastases from breast cancer were obtained from patients that received bone metastasis resection at Changzheng hospital (Shanghai, China). All of the subjects provided written informed consent. Ethical consent was granted by the Committees for Ethical Review of Research involving Human Subjects of Second Military Medical University (Shanghai, China).

**RNA isolation and Real-time RT-PCR**

For quantitative miR-124 PCR assays, total RNA was isolated using the mirVana miRNA isolation kit (Applied Biosystems Inc., Foster City, CA, USA) according to the manufacturer’s protocol. RNA was then reverse-transcribed to cDNA using the PrimeScript™ RT reagent Kit (Takara, Dalian, China) with a miR-124 specific stem-loop RT primer and analyzed with SYBR Green-based real-time PCR (Takara). To detect mRNA expression levels, total RNA was isolated using TRIZOL (Invitrogen, Carlsbad, CA, USA) per the manufacturer’s protocol, reverse-transcribed with SuperScript III (Invitrogen) using random primers, and analyzed with SYBR Green-based real-time PCR. Gene expression was analyzed using the 2^-ΔΔCT^ method. miRNA transcript was normalized against U6, and mRNA expression was normalized against β-actin. At least three independent experiments were performed for each condition. Primer sequences are listed in Supplementary Table 3.

**In situ hybridization (ISH)**

Fluorescence ISH was used to detect miR-124 expression in tissue microarray slides containing 79 paired primary breast cancer tissues and adjacent non-tumorous mammary tissues as well as 34 bone metastases from breast cancer. ISH was performed using a miR-124 locked nucleic acid probe (5′-digoxigenin-GGCATTCACCGCGTGCCTTA-3′-digoxigenin) and the microRNA ISH Optimization Kit (Exiqon, Vedbaek, Denmark) according to the manufacturer’s instructions as described previously^[2]^. The signals were examined with a BX51 fluorescence microscope (Olympus) and quantified using the Aperio Spectrum^®^ software with a pixel count algorithm.

**Construction of lentivirus**

To construct the lenti-miR-124 plasmid (pLenO-DCE-Puro-miR-124), cDNA encoding pri-miR-124 was appended with EcoRI and BamHI sites and cloned into pLenO-DCE-Puro Vector (Bio-link, Shanghai, China). The backbone plasmid expressing Green Fluorescent Protein (GFP) was used as a negative control (NC). Lentiviruses were produced by four-plasmid cotransfection of 293T cells with the packaging helper plasmid pRSV-Rev, pMDLg/pRRE, pMD2.G and pLenO-DCE (Transfer Vector). The viruses were concentrated by ultracentrifugation. Viral titers were determined by infection of 293T cells with serial dilutions of the vector stock. Forty-eight hours after infection, the number of GFP positive cells was scored by fluorescence-activated cell sorting (FACS) analysis to determine the titer. The lentivirus carrying miR-124 inhibitor and its negative control (NC) were constructed by Obio Technology (Shanghai, China) according to the method described previously^[3]^. TuD-miR-124 fragment containing two miR-124 binding sequence or its control fragment was synthesized and cloned into the plasmid pLKD-CMV-DsRed2-U6-shRNA digested with AgeI and EcoRI.

**Animal models**

To investigate the effect of miR-124 on breast cancer cell survival in the bone microenvironment, luciferase-labeled MDA-MB-231 cells (2 × 10^5^) infected with lentivirus expressing miR-124 or NC were transplanted into female Balb/c nude mice via the intratibia route (N = 11 / group). Luciferase signals were detected in tibias using the IVIS200 (Caliper LS, Hopkinton, MA, USA) imaging systems 4 weeks after injection. Cancer cell-induced osteolysis was detected by X-ray analysis.

To investigate the effect of miR-124 on the bone metastasis of breast cancer cells *in vivo*, luciferase-labeled MDA-MB-231 cells infected with lentivirus expressing miR-124 or NC (1 × 10^5^), as well as luciferase-labeled MCF7 cells infected with lentivirus expressing miR-124 inhibitor or NC (1 × 10^5^) were inoculated into the left ventricle of Balb/c nude mice (N = 8 / group, N = 5 / group, respectively). Mice were monitored with an *ex vivo* imaging system. Metastases were confirmed by X-ray analysis and hematoxylin-eosin (H&E) staining.

To explore if miR-124 can prevent bone metastasis, luciferase-labeled MDA-MB-231 cells (1 × 10^5^) were inoculated into the left ventricle of Balb/c nude mice (N = 6 / group). When bioluminescence was visible, mice were treated with injections of 10 nmol ago-miR-124 (miR400004422, RiboBio, Guangzhou, China) or NC (miR04201) via tail vein twice a week for up to four weeks and sacrificed one week after the last treatment. Bone metastases were analyzed by X-ray and histopathologically confirmed with H&E staining.

To determine the role of IL-11 in the suppression of bone metastasis by miR-124, MCF7 cells (1 × 10^5^) infected with lentivirus expressing miR-124 inhibitor or NC were firstly inoculated into the left ventricle of Balb/c nude mice (N = 6 / group), and then 5μg IL-11 neutralizing antibody or the control IgG were injected into the tail veins of the mice twice a week for up to three weeks in both groups. Mice were monitored with an *ex vivo* imaging system.

All of the mouse experiments were performed according to protocols reviewed and approved by the Institutional Animal Care and Use Committee at the Second Military Medical University.

**Transfection of miR-124 mimic or miR-124 inhibitor and collection of conditioned media**

miR-124 mimic and negative control (NC) or miR-124 inhibitor and inhibitor NC were purchased from RiboBio (RibiBio, Guangzhou, China). MDA-MB-231 cells were transfected with miR-124 mimic and NC, and MCF7 cells were transfected with miR-124 inhibitor and inhibitor NC using Lipofectamine2000 (Invitrogen) according to the manufacturer’s instructions. Twenty-four hours after transfection, the media from MDA-MB-231 cells transfected with miR-124 mimic or NC and the media from MCF7 cells transfected with miR-124 inhibitor or inhibitor NC were collected as conditioned media.

**Proliferation and differentiation of BMMs**

BMMs (5 × 10^3^ cells / well) were seeded into 96-well plate and incubated with M-CSF (10 ng/ml) and the receptor activator of nuclear factor-κB ligand (RANKL) (50 ng/ml) in the presence of 30% conditioned media, which was replaced every 48 h. The proliferation of BMM cells was analyzed using Cell Counting Kit-8 (Dojinodo, Shanghai, China) according to the manufacturer’s protocol. After 5 - 7 days, BMM differentiation was analyzed by tartrate resistant acid phosphatase (TRAP) staining and actin-ring formation assays. For TRAP staining, the cells were fixed and stained using the TRAP activity kit (Sigma-Aldrich, St. Louis, MO, USA), and TRAP-positive multinucleated cells containing three or more nuclei were counted as mature osteoclasts. For actin-ring formation assay, cells were fixed with 4% PFA in phosphate-buffered saline (PBS) for 10 min. The cells were permeabilized with 0.1% Triton-X 100 in PBS for 5 min and incubated with rhodamine-conjugated phalloidin (Molecular Probes, Eugene, OR, USA) to visualize F-actin.

**Western blot**

Proteins were extracted using RIPA buffer (P0013B, Beyotime, Suzhou, China) supplemented with protease inhibitor cocktail (Merck & Co., Kenilworth, NJ, USA), separated by SDS-PAGE and transferred to a nitrocellulose membrane (HAHY00010, EMD Millipore, Billerica, MA, USA). The membrane was probed with IL-11 antibody (sc-7924, Santa Cruz, CA, USA) overnight at 4 °C, followed by incubation with a donkey-anti-rabbit secondary antibody IRDye 800 (LI-COR, Lincoln, NE, USA). Detection was performed using an Odyssey IR imaging system (LI-COR). Protein was semi-quantified using BandScan (Version 5.0).

**Immunohistochemical (IHC) staining**

Formalin-fixed paraffin-embedded sections were deparaffinized by xylene and rehydrated by graded alcohol. Endogenous peroxidase was blocked by 3% H2O2 followed by antigen retrieval. Slides were blocked in 10% goat serum for two hours at room temperature, incubated with IL-11 antibody (sc-7924) overnight at 4 °C and incubated with the secondary antibody at room temperature for 30 min. The staining was developed using EnVision Detection Rabbit / Mouse Kit (GK500710, GeneTech, Shanghai, China).

**Construction of luciferase reporter plasmids and luciferase reporter assays**

To construct the luciferase reporter plasmid encoding IL-11 3’untranslated regions (3′UTR), a 1562 bp fragment of the 3′UTR from human IL-11 was sub-cloned into the psiCHECK2 vector (Promega, Madison, WI, USA) using XhoI and NotI restriction sites. Mutation of the miR-124 binding site on the IL-11 3′UTR reporter vector was performed using overlap extension by PCR as described previously^[4]^. All of the vectors were verified by sequencing. Primer sequences are listed in Supplementary Table 4.

For the luciferase reporter assays, HEK293 cells cultured in a 24-well plate were co-transfected with 20 pmol/well miR-124 mimic or NC and 400 ng/well psiCHECK2 plasmids using 2 μl/well Lipofectamine2000 (Invitrogen). Forty-eight hours after transfection, Renilla and firefly luciferase activities were measured with the Dual-Luciferase Reporter Assay (Promega, Madison, WI, USA) using a luminometer (Synergy™ 4 Hybrid Microplate Reader, BioTek, Winooski, VT, USA). The luciferase score was calculated by normalizing the Renilla luciferase signal against the firefly signal. At least three independent experiments were performed for each condition.

**Reagent**

Human IL-11 neutralizing antibody (AF-218-NA), normal goat IgG control antibody (AB-108-C) and recombinant human IL-11 protein (218-IL-005) were purchased from R&D systems (Minneapolis, MN, USA).

**Statistics**

All statistical analyses were performed using SPSS version 21.0 software. Statistical tests for data analysis included two-tailed Student’s t test, log-rank test, Mann-Whitney U test, Spearman correlation analysis, and Fisher’s exact test. A *P* value <0.05 was considered statistically significant.

References

1. Ng PK, Tsui SK, Lau CP, Wong CH, Wong WH, Huang L, et al. CCAAT/enhancer binding protein beta is up-regulated in giant cell tumor of bone and regulates RANKL expression. J Cell Biochem. 2010;110:438-46.
2. Zhou W, Yin H, Wang T, Liu T, Li Z, Yan W, et al. MiR-126-5p regulates osteolysis formation and stromal cell proliferation in giant cell tumor through inhibition of PTHrP. Bone. 2014;66:267-76.
3. Hollensen AK, Bak RO, Haslund D, Mikkelsen JG. Suppression of microRNAs by dual-targeting and clustered Tough Decoy inhibitors. RNA Biol. 2013;10:406-14.
4. Ho SN, Hunt HD, Horton RM, Pullen JK, Pease LR. Site-directed mutagenesis by overlap extension using the polymerase chain reaction. Gene. 1989;77:51-9.

**Supplementary Figures**

**
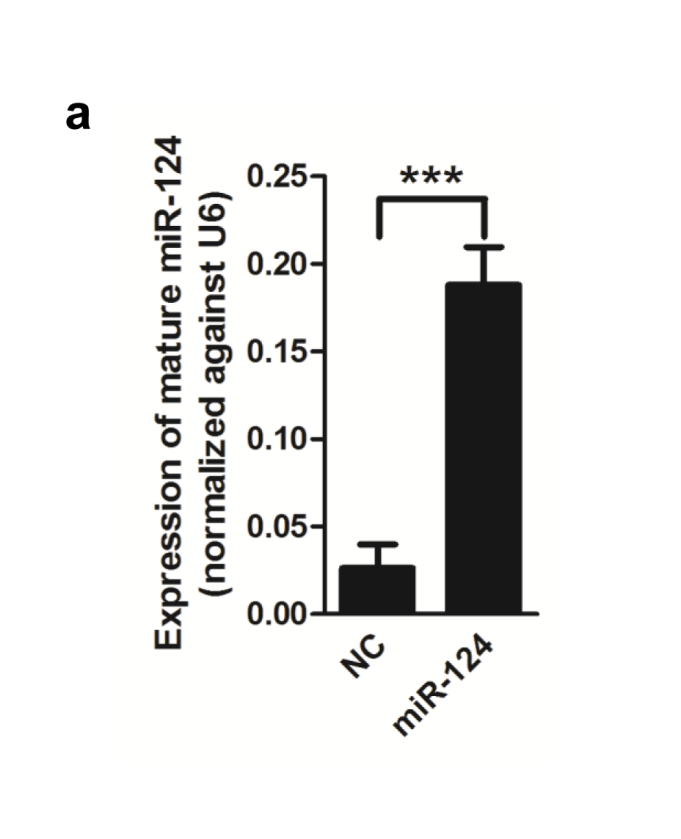
**

**Supplementary Figure 1. Mature miR-124 is elevated in MDA-MB-231 cells infected with miR-124 lentivirus.**

**(a)** Real-time PCR quantification of miR-124 expression in MDA-MB-231 cells infected with miR-124 lentivirus (miR-124) and in control cells (NC). Expression of miR-124 was normalized against an endogenous control U6. *** *P* < 0.001 by two-tailed Student’s t test. Experiments were performed in triplicate and data are shown as mean ± SD.


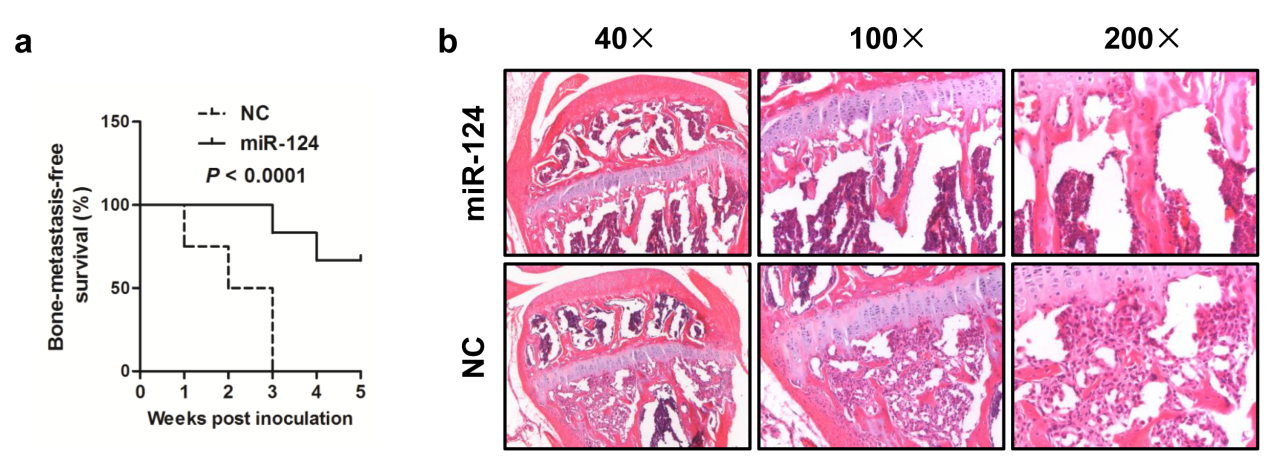


**Supplementary Figure 2. Restoration of miR-124 suppresses bone metastasis of MDA-MB-231 cells *in vivo*.**

**(a)** The bone metastasis free survival of mice transplanted with MDA-MB-231 cells infected with lentivirus expressing miR-124 (miR-124) or control virus (NC) was analyzed by the Kaplan-Meier method and compared using the log-rank test.

**(b)** Tibias were collected from mice inoculated with luciferase-labeled MDA-MB-231 cells stably expressing miR-124 or NC and sectioned for H&E at an original magnification of ×40 (Left). Tumor-bone interface is shown at an original magnification of ×200 (Right).


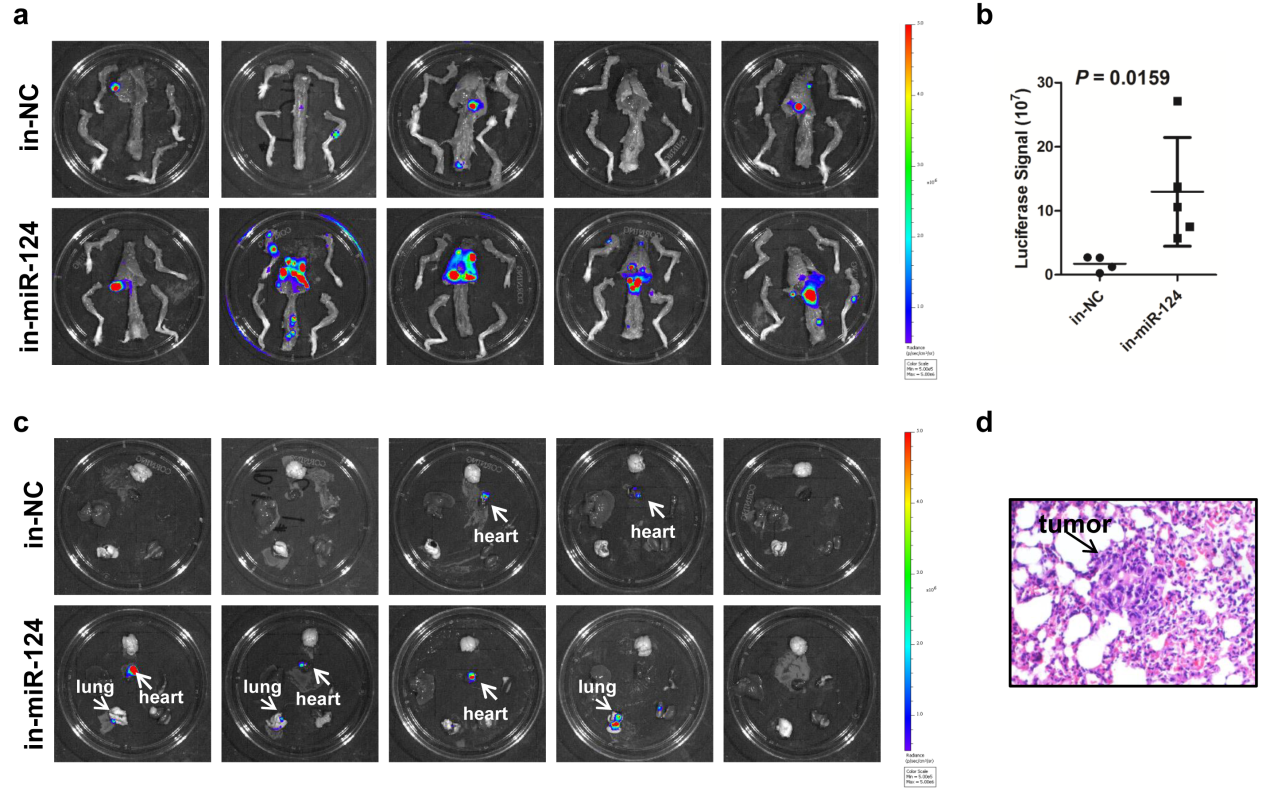


**Supplementary Figure 3. Inhibition of miR-124 promotes bone metastasis and lung metastasis of MCF7 cells *in vivo*.**

**(a)** Images of luciferase signal in the bones from mice inoculated with luciferase-labeled MCF7 cells stably expressing miR-124 inhibitor (in-miR-124) or negative control (in-NC) into the left ventricle.

**(b)** Statistical analysis showed increased luciferase signal in mice inoculated with luciferase-labeled MCF7 cells stably expressing miR-124 inhibitor. Horizontal line indicates median value. *P* = 0.0159 by Mann-Whitney U test.

**(c)** Images of luciferase signal in organs including brain, heart, lungs, liver, spleen, kidneys and gut from mice inoculated with luciferase-labeled MCF7 cells stably expressing miR-124 inhibitor (in-miR-124) or negative control (in-NC) into the left ventricle. The arrow indicates luciferase signal in the lungs and hearts.

**(d)** The lungs from mice inoculated with luciferase-labeled MCF7 cells stably expressing miR-124 inhibitor were collected and sectioned for H&E staining at an original magnification of ×200.
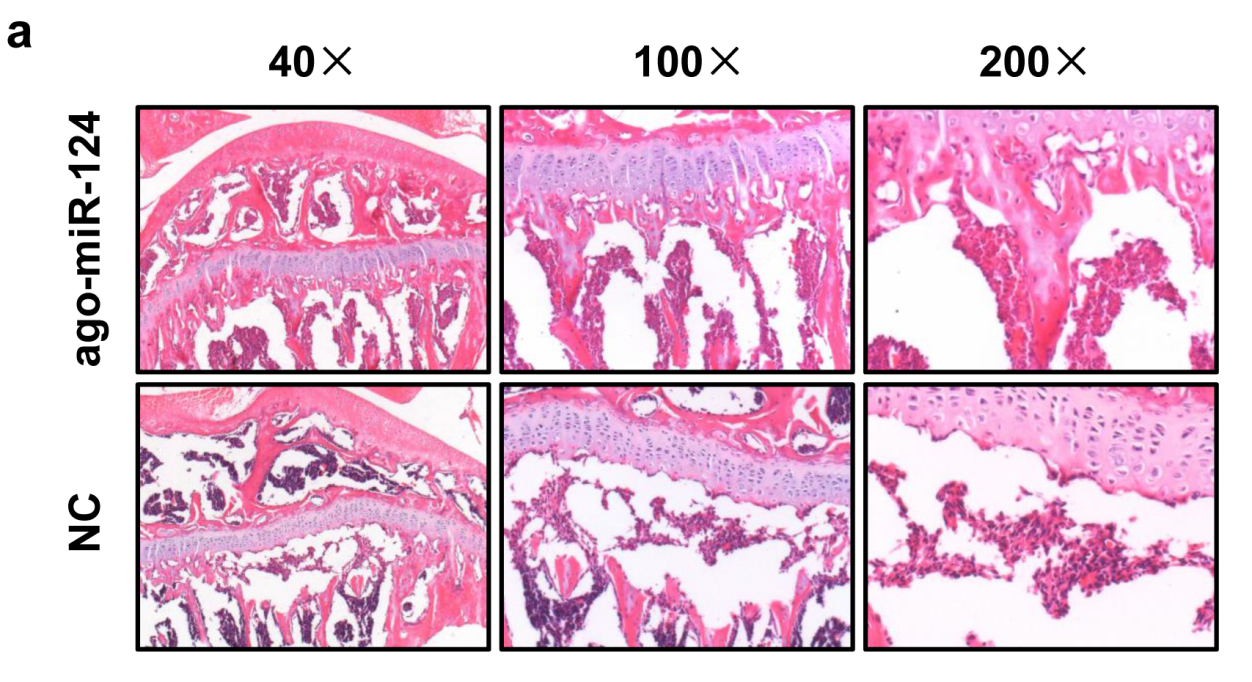


**Supplementary Figure 4. H&E staining of tibias from mice inoculated with MDA-MB-231 cells and then injected with ago-miR-124 or NC.**

**(a)** Mouse tibias were collected and sectioned from mice inoculated with MDA-MB-231 cells and then injected with ago-miR-124 or NC. H&E staining shows the tumor-bone interface at an original magnification of ×40, ×100 and ×200.


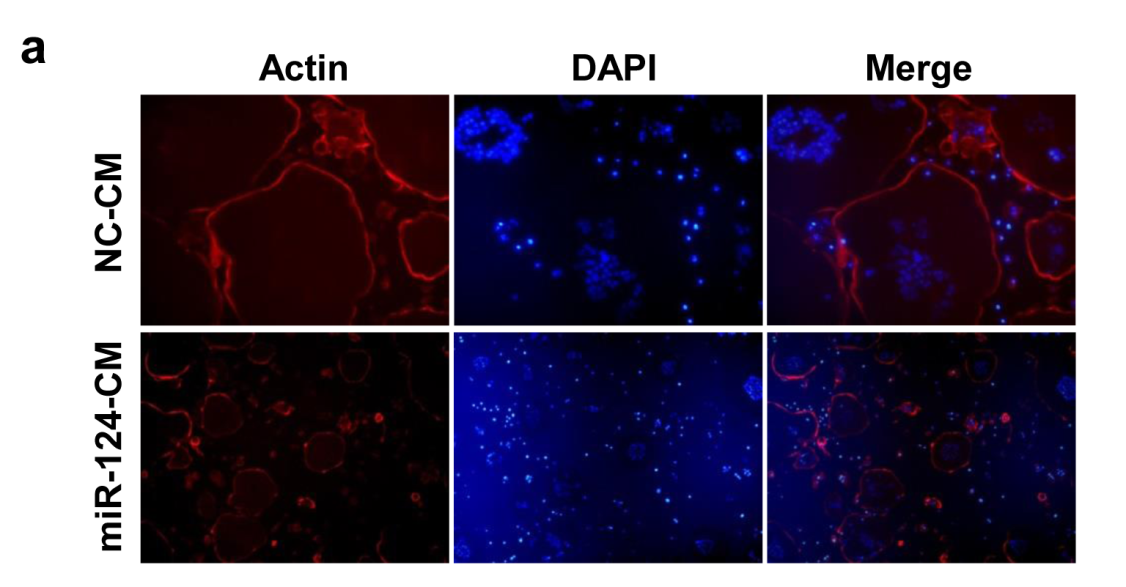


**Supplementary Figure 5. miR-124-CM also reduced the formation of actin-ring structures in BMMs.**

**(a)** BMMs were first cultured with conditioned media from MDA-MB-231 transfected with miR-124 mimic (miR-124-CM) or NC (NC-CM), then were fixed and stained for F-actin to show the actin-rings formation at an original magnification of ×40.


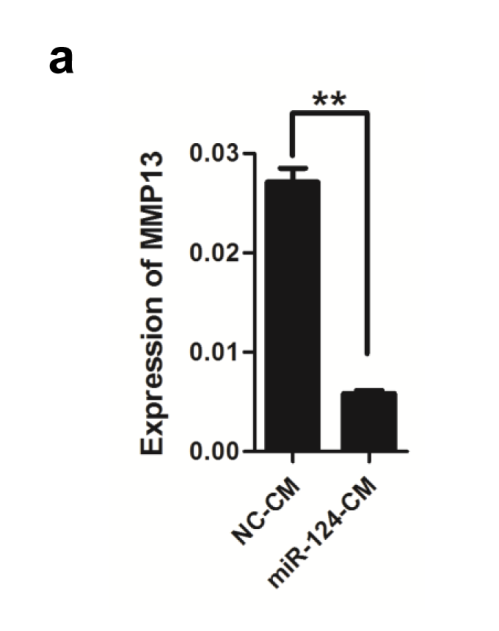


**Supplementary Figure 6. The expression of MMP13 in MC-3T3E1 cells cultured with miR-124 conditioned medium or NC conditioned medium.**

**(a)** Real-time RT-PCR analysis of expression of MMP13 in MC-3T3E1 cells treated with miR-124-CM or NC-CM. Gene expression was normalized against β-actin. ** *P* < 0.01 by two-tailed Student’s t test. Experiments were performed in triplicate and data are shown as mean ± SD.


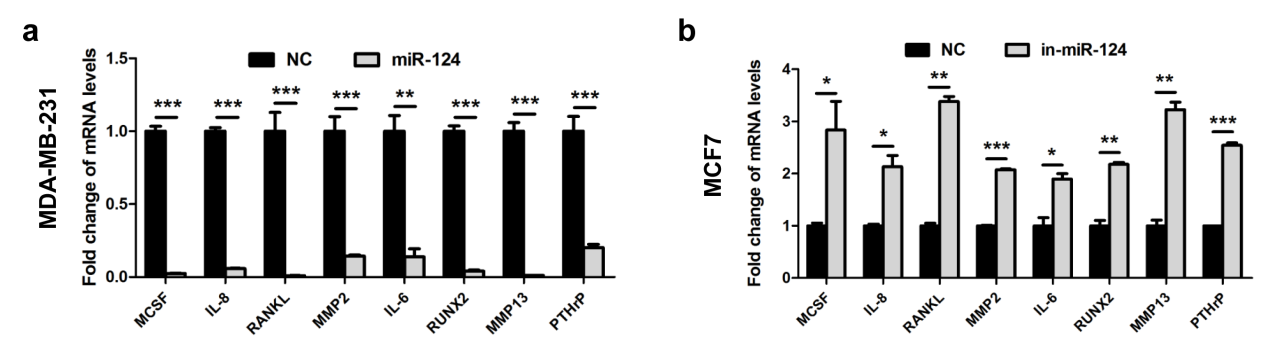


**Supplementary Figure 7. miR-124 inhibits the expression of several cancer cell-derived osteoclast-activating factors.**

Real-time RT-PCR was used to detect the expression of MCSF, IL-6, IL-8, RANKL, MMP2, MMP13, RUNX2 and PTHrP in MDA-MB-231 cells transfected with miR-124 mimic (miR-124) or NC **(a)** and MCF7 cells transfected with miR-124 inhibitor (in-miR-124) or inhibitor NC (NC) **(b)**. ** P* < 0.05, ** *P* < 0.01 and *** *P* < 0.001 by two-tailed Student’s t test. Experiments were performed in triplicate and data are shown as mean ±SD.


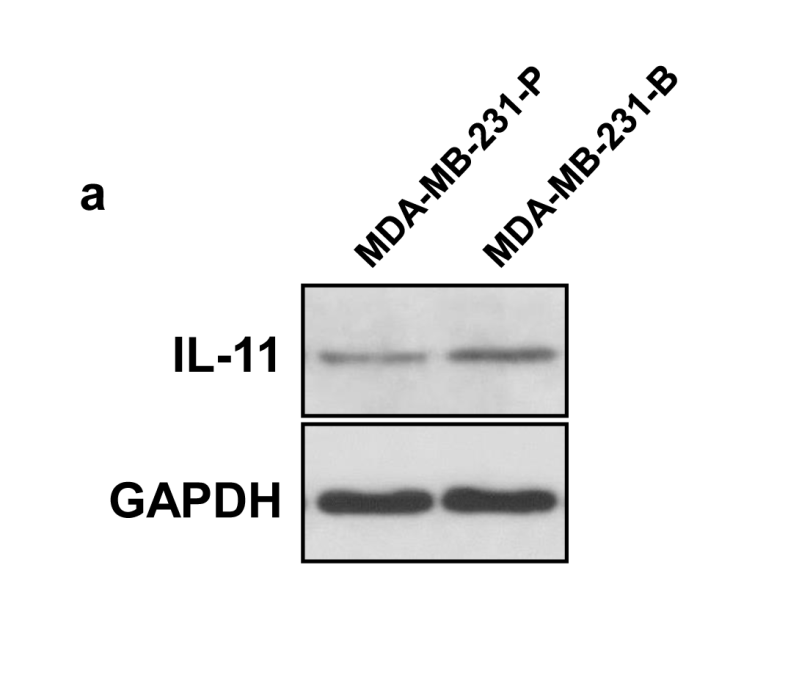


**Supplementary Figure 8. IL-11 expression in parental MDA-MB-231 cells and the highly bone metastatic variant.**

**(a)** IL-11 protein expression as determined by Western blot was obviously enhanced in the highly bone metastatic MDA-MB-231 cells variant (MDA-MB-231-B) as compared with parental MDA-MB-231 cells (MDA-MB-231-P).


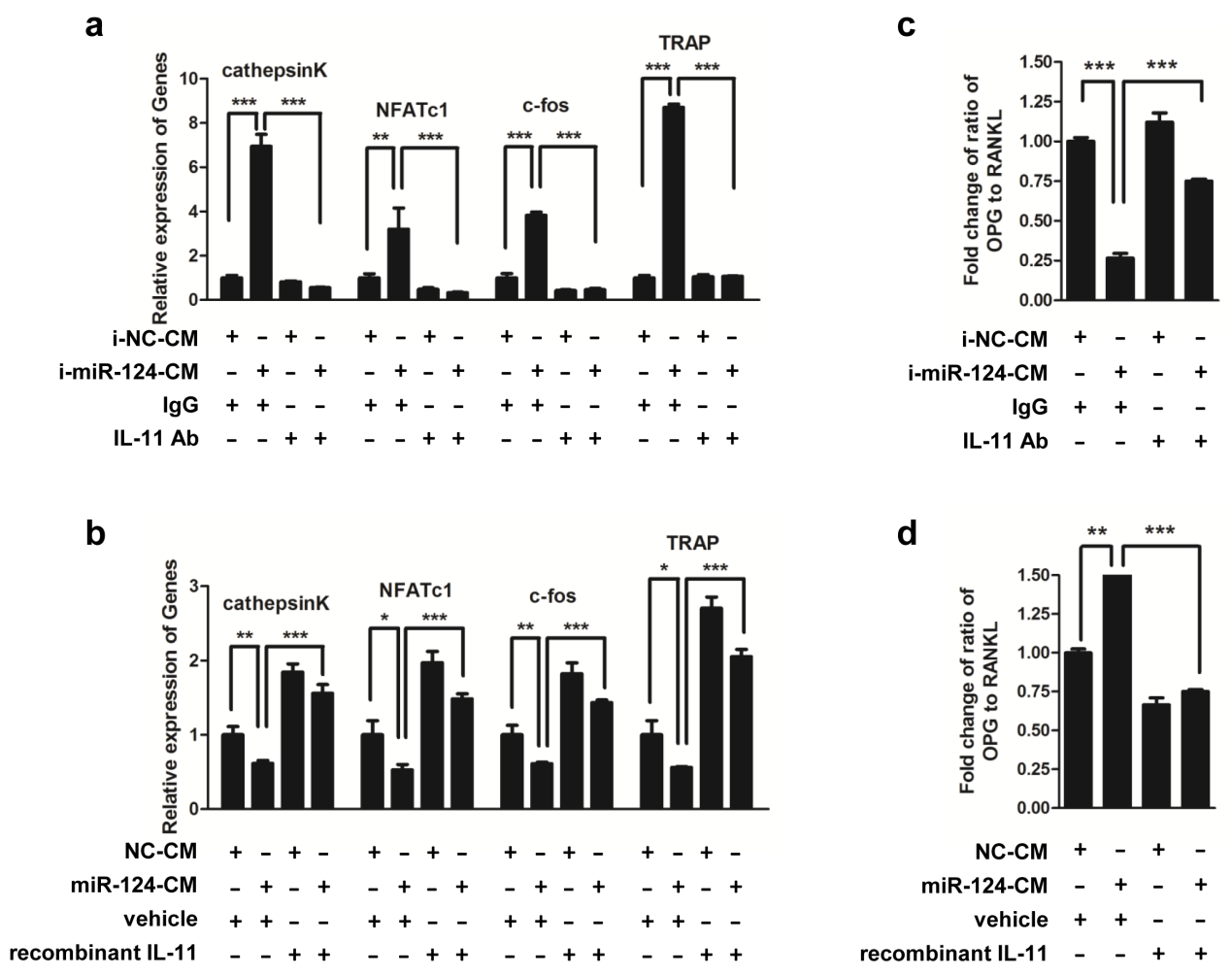


**Supplementary Figure 9. IL-11 partially reverses the effects of miR-124 on osteoclasts and osteoblasts.**

**(a**) IL-11 neutralizing antibody treatment (5 μg/ml) efficiently reversed the promoting effects of conditioned medium from MDA-MB-231 cells transfected with miR-124 inhibitor (i-miR-124-CM) on the expression of Cathepsin K, NFATc1, c-fos and TRAP in RAW264.7 cells.

**(b)** Recombinant human IL-11 treatment (10 ng/ml) partially reversed the suppressive effects of conditioned medium from MDA-MB-231 cells transfected with miR-124 mimic (miR-124-CM) on the expression of Cathepsin K, NFATc1, c-fos and TRAP in RAW264.7 cells.

**(c)** Suppressive effect of i-miR-124-CM on the ratio of OPG to RANKL in MC3T3-E1 cells was substantially inhibited upon the treatment of IL-11 neutralizing antibody.

**(d)** Promoting effect of miR-124-CM on the ratio of OPG to RANKL in MC3T3-E1 cells was partially reversed upon the treatment of recombinant human IL-11.

** P* < 0.05, ** *P* < 0.01 and *** *P* < 0.001 by two-tailed Student’s t test. Experiments were performed in triplicate and data are shown as mean ±SD.


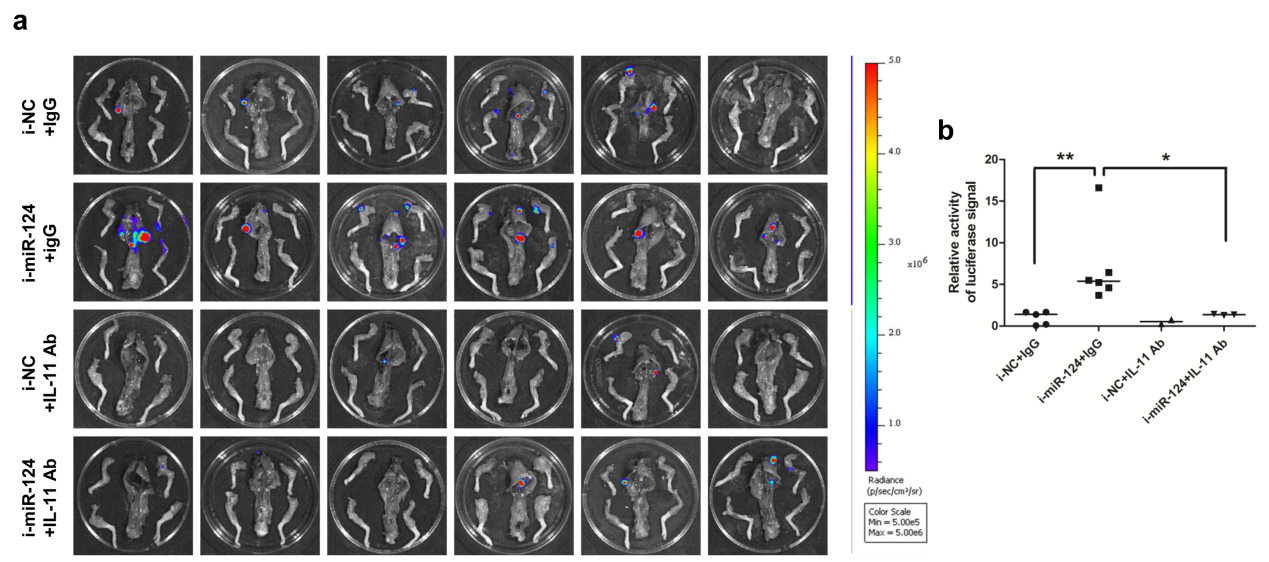


**Supplementary Figure 10. IL-11 neutralizing antibody partially reverses the promoting effect of miR-124 inhibitor on the bone metastasis of breast cancer cells in mice.**

**(a)** MCF7 cells stably expressing miR-124 inhibitor (i-miR-124) or NC (i-NC) were firstly inoculated into the left ventricle of nude mice, and then 5μg IL-11 neutralizing antibody (IL-11 Ab) or the control IgG (IgG) were injected into the tail veins of the mice. The bones including femurs, ribs, spine and tibias of mice from these four groups were collected and luciferase signals were measured with an *ex vivo* imaging system.

**(b)** Statistical analysis showed IL-11 neutralizing antibody partially reverses the promoting effect of miR-124 inhibitor on the bone metastasis of MCF7 cells. Horizontal line indicates median value. ** P* < 0.05, ** *P* < 0.01 by Mann-Whitney U test.


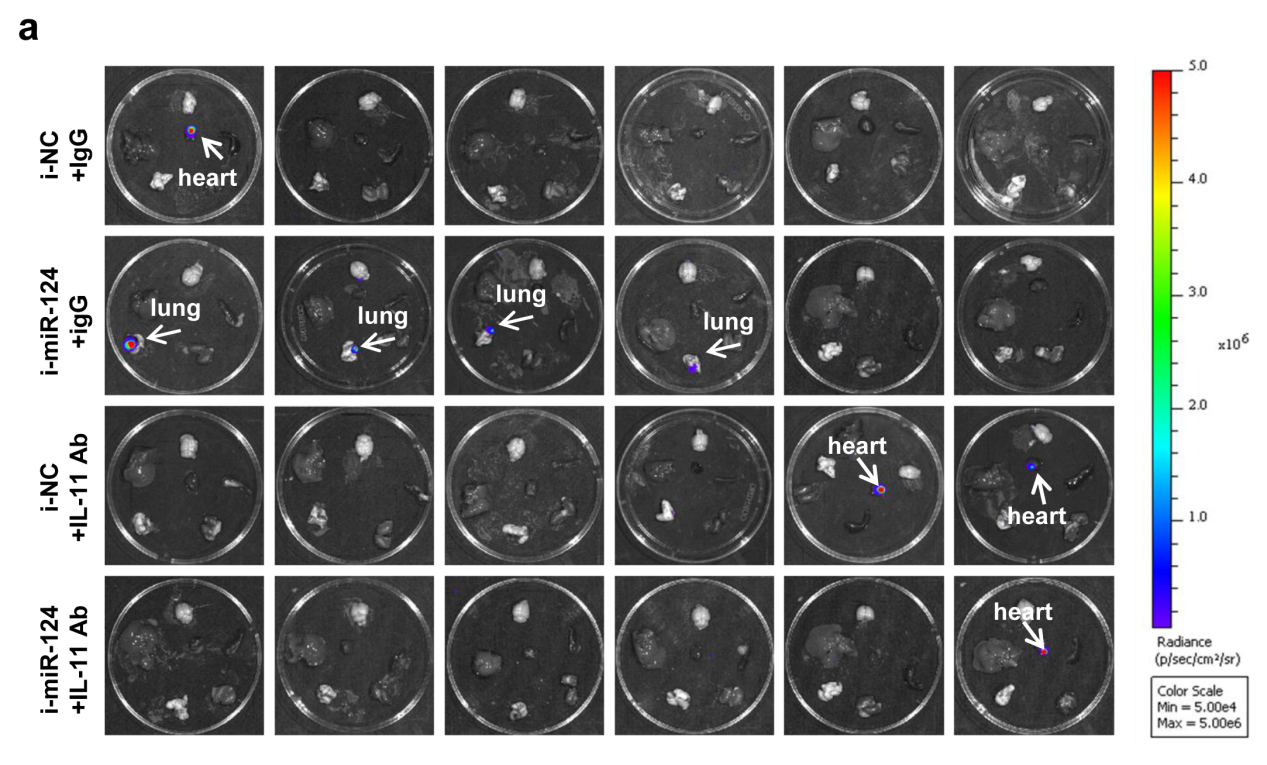


**Supplementary Figure 11. IL-11 neutralizing antibody reverses the promoting effect of miR-124 inhibitor on the lung metastasis of breast cancer cells in mice.**

**(a)** MCF7 cells stably expressing miR-124 inhibitor (i-miR-124) or NC (i-NC) were firstly inoculated into the left ventricle of nude mice, and then 5μg IL-11 neutralizing antibody (IL-11 Ab) or the control IgG (IgG) were injected into the tail veins of the mice. The organs including brain, lungs, liver, spleen, kidneys and gut of mice from these four groups were collected and luciferase signals were measured with an *ex vivo* imaging system.


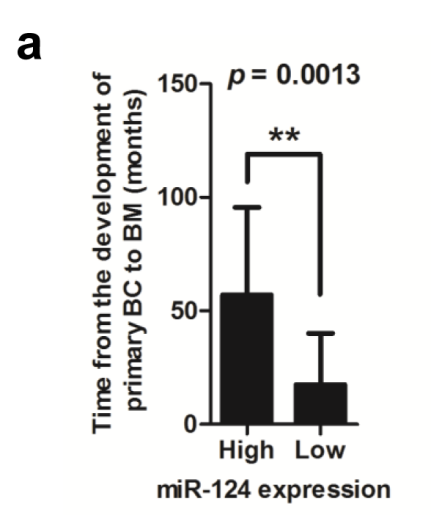


**Supplementary Figure 12. Time from the development of primary breast cancer to bone metastasis was longer in patients with high miR-124 expression in bone tissues than that in patients with low miR-124 expression in bone tissues.**

**(a)** A comparison of the time from primary cancer surgery to bone metastasis development between the patients with high and low miR-124 expression. The median value of miR-124 in all 34 samples was chosen as the cut-off point for separating bone metastasis tissues with high expression of miR-124 from tissues with low level of miR-124. *P* = 0.0013 by Mann-Whitney U test.


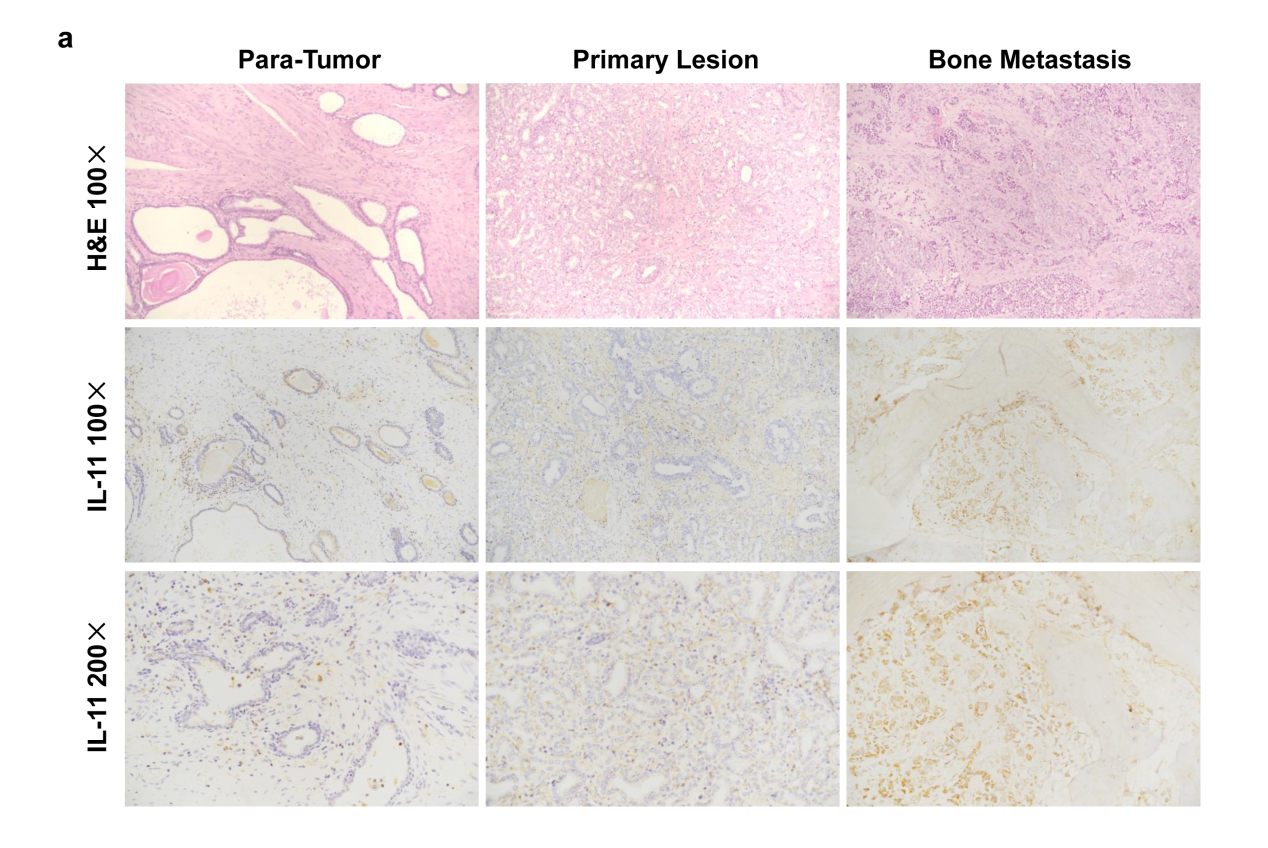


**Supplementary Figure 13. IL-11 expression in the human primary breast cancer tissues, para-tumor tissues and bone metastasis tissues.**

**(a)** IHC analysis was used to determine the expression of IL-11 in paired primary breast cancer lesions (Primary Lesion) and para-tumorous mammary tissues (Para-Tumor) as well as bone metastasis tissues (Bone Metastasis) of breast cancer. Representative images of H&E staining were shown on the top at an original magnification of ×100 while representative images of IHC analysis were shown in the middle at an original magnification of ×100 and on the bottom at an original magnification of ×200.


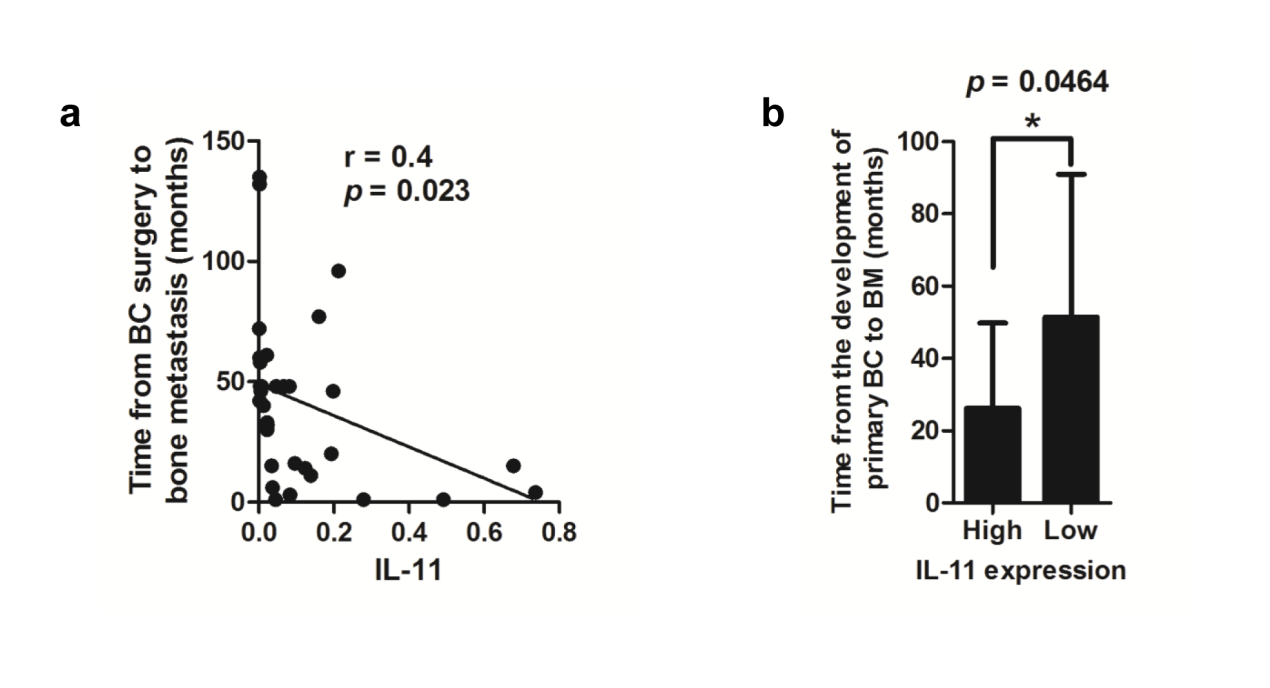


**Supplementary Figure 14. IL-11 expression in bone metastasis tissues was negatively related to the time from the development of primary breast cancer to bone metastasis.**

**(a)** A correlation analysis was performed between IL-11 expression as detected by IHC and the time from the development of primary breast cancer to bone metastasis. r = 0.4, *p* = 0.023 by linear regression.

**(b)** A comparison of the time from the development of primary cancer to bone metastasis between the patients with high and low IL-11 expression. The median value of IL-11 in all 34 samples was chosen as the cut-off point for separating bone metastasis tissues with high expression of IL-11 from tissues with low level of IL-11. *P* = 0.0464 by Mann-Whitney U test.

**
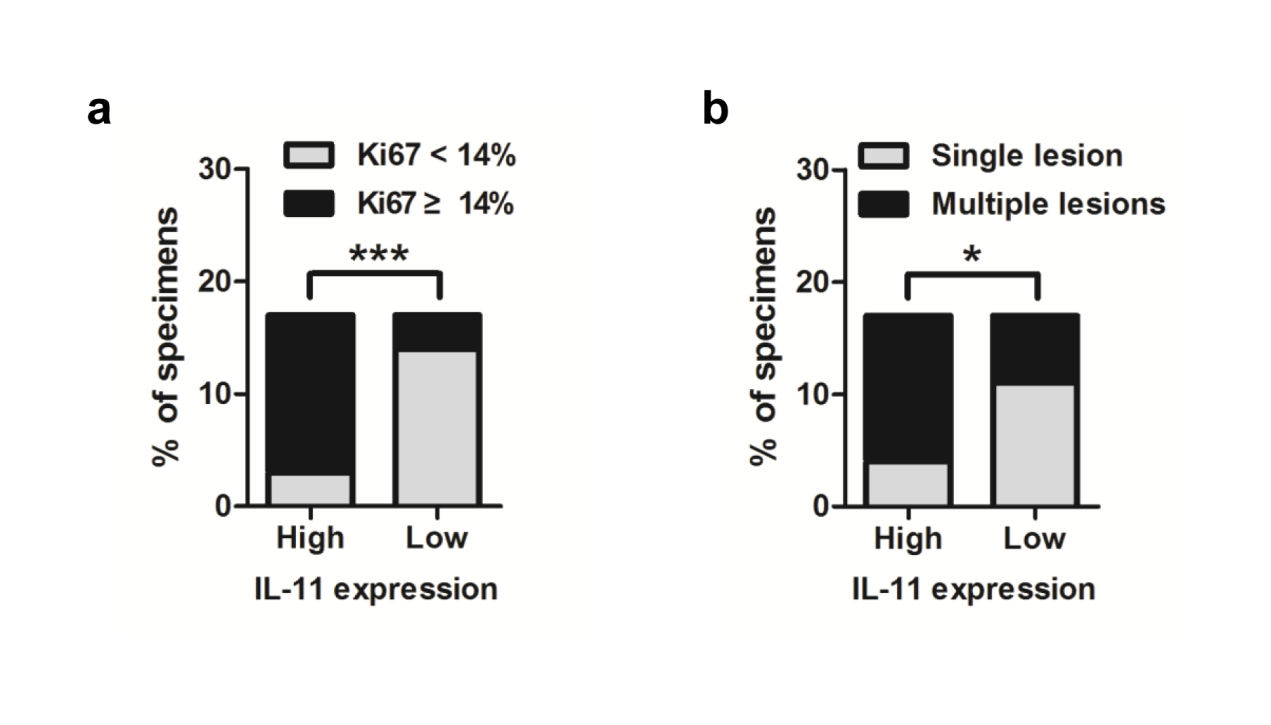
**

**Supplementary Figure 15. IL-11 level was correlated to the clinicopathological characteristics of breast cancer patients with bone metastasis.**

**(a)** Percentages of specimens with low or high IL-11 expression were relative to percentages of specimens with Ki67 < 14% or Ki67 ≥ 14%.

**(b)** Percentages of specimens with low or high IL-11 expression were relative to percentages of specimens with single bone lesion or multiple bone lesions.

**Supplementary Tables**

**Supplementary Table 1. miR-124 expression and clinicopathological characteristics of breast cancer patients with bone metastasis.**

| **miR-124** | | | | | |
| --- | --- | --- | --- | --- | --- |
|  | **All cases High expression Low expression** | | |  | |
| **Variables** | **(n=34)** | **(n=17)** | **(n=17)** | ***p* Value*** | |
| **Age (years)** | | | | | |
| > 53.5† | 17 | 7 | 10 | | 0.4935 |
| ≤ 53.5 | 17 | 10 | 7 | |  |
| **ER status** | | | | | |
| Positive | 16 | 10 | 6 | | 0.3028 |
| Negative | 18 | 7 | 11 | |  |
| **PR status** | | | | | |
| Positive | 16 | 11 | 5 | | 0.0844 |
| Negative | 18 | 6 | 12 | |  |
| **HER2 status** | | | | | |
| Positive | 17 | 10 | 5 | | 0.1663 |
| Negative | 17 | 7 | 12 | |  |
| **Ki67** |  |  |  | |  |
| < 14% | 17 | 12 | 5 | | 0.0381 |
| ≥ 14% | 17 | 5 | 12 | |  |
| **Preoperative KPS** | | | | | |
| ≥80 | 13 | 10 | 3 | | 0.0324 |
| ＜80 | 21 | 7 | 14 | |  |
| **Preoperative Frankel** | | | | | |
| ABC | 20 | 9 | 11 | | 0.7283 |
| DE | 14 | 8 | 6 | |  |
| **Preoperative ambulation** | | | | | |
| Yes | 14 | 8 | 6 | | 0.7283 |
| No | 20 | 9 | 11 | |  |
| **Number of bone lesions** | | | | | |
| Single | 15 | 11 | 4 | | 0.0366 |
| Multiple | 19 | 6 | 13 | |  |
| **Postoperative KPS** | | | | | |
| ≥80 | 28 | 15 | 13 | | 0.6562 |
| ＜80 | 6 | 2 | 4 | |  |
| **Postoperative Frankel** | | | | | |
| ABC | 4 | 1 | 3 | | 0.6012 |
| DE | 30 | 16 | 14 | |  |
| **Postoperative ambulation** | | | | | |
| Yes | 30 | 16 | 14 | | 0.6012 |
| No | 4 | 1 | 3 | |  |

The median value of all 34 samples was chosen as the cut-off point.

***** Fisher’s exact test.

† Mean age.

Abbreviations: ER, Estrogen receptor; PR, Progesterone receptor; HER2, human epidermal growth factor receptor type 2; KPS, Karnofsky performance score.

**Supplementary Table 2. IL-11 expression and clinicopathological characteristics of breast cancer patients with bone metastasis.**

| **IL-11** | | | | | |
| --- | --- | --- | --- | --- | --- |
|  | **All cases High expression Low expression** | | |  | |
| **Variables** | **(n=34)** | **(n=17)** | **(n=17)** | ***p* Value*** | |
| **Age (years)** | | | | | |
| > 53.5† | 17 | 9 | 8 | | 1 |
| ≤ 53.5 | 17 | 8 | 9 | |  |
| **ER status** | | | | | |
| Positive | 16 | 9 | 7 | | 0.7319 |
| Negative | 18 | 8 | 10 | |  |
| **PR status** | | | | | |
| Positive | 16 | 6 | 10 | | 0.3028 |
| Negative | 18 | 11 | 7 | |  |
| **HER2 status** | | | | | |
| Positive | 15 | 8 | 7 | | 1 |
| Negative | 19 | 9 | 10 | |  |
| **Ki67** |  |  |  | |  |
| < 14% | 17 | 3 | 14 | | 0.0004 |
| ≥ 14% | 17 | 14 | 3 | |  |
| **Preoperative KPS** | | | | | |
| ≥80 | 13 | 4 | 9 | | 0.1571 |
| ＜80 | 21 | 13 | 8 | |  |
| **Preoperative Frankel** | | | | | |
| ABC | 20 | 11 | 9 | | 0.7283 |
| DE | 14 | 6 | 8 | |  |
| **Preoperative ambulation** | | | | | |
| Yes | 14 | 6 | 8 | | 0.7283 |
| No | 20 | 11 | 9 | |  |
| **Number of bone lesions** | | | | | |
| Single | 15 | 4 | 11 | | 0.0366 |
| Multiple | 19 | 13 | 6 | |  |
| **Postoperative KPS** | | | | | |
| ≥80 | 28 | 13 | 15 | | 0.6562 |
| ＜80 | 6 | 4 | 2 | |  |
| **Postoperative Frankel** | | | | | |
| ABC | 4 | 2 | 2 | | 1 |
| DE | 30 | 15 | 15 | |  |
| **Postoperative ambulation** | | | | | |
| Yes | 17 | 15 | 15 | | 1 |
| No | 17 | 2 | 2 | |  |

The median value of all 34 samples was chosen as the cut-off point.

***** Fisher’s exact test.

† Mean age.

**Supplementary Table 3. Sequences of primers used in Real-time RT-PCR**

|  | Sequences (5′-3′) |
| --- | --- |
| U6 RT primer  U6 forward | AAAATATGGAACGCTTCACGAATTTG  CTCGCTTCGGCAGCACATATACT |
| U6 reserve | ACGCTTCACGAATTTGCGTGTC |
| hsa-miR-124  RT primer | CTCAACTGGTGTCGTGGAGTCGGCAATTCAGTTGAGTTGGCATT |
| hsa-miR-124-3p forward | TCGGCAGGTAAGGCACGCGGTG |
| hsa-miR-124-3p reverse | TCAACTGGTGTCGTGGAGTCGGC |
| mouse cathepsinK forword | AAGTGGTTCAGAAGATGACGGGAC |
| mouse cathepsinK reverse | TCTTCAGAGTCAATGCCTCCGTTC |
| mouse NFATc1 forward | TGGAGAAGCAGAGCACAGAC |
| mouse NFATc1 reverse | GCGGAAAGGTGGTATCTCAA |
| mouse c-fos forward | CAAGCGGAGACAGATCAACTTG |
| mouse c-fos reverse | TTTCCTTCTCTTTCAGCAGATTGG |
| mouse TRAP forward | GCTGGAAACCATGATCACCT |
| mouse TRAP reverse | GAGTTGCCACACAGCATCAC |
| mouse OPG forward | CAGATGGGTTCTTCTCAGGT |
| mouse OPG reverse | TCTCGGCATTCACTTTGGTC |
| mouse RANKL forward | ATCCCATCGGGTTCCCATAA |
| mouse RANKL reverse | TTCGTGCTCCCTCCTTTCAT |
| mouse MMP13 forward | ACTACCATCCTGCGACTCTTG |
| mouse MMP13 reverse  mouse β-actin forward | GTTTGCCAGTCACCTCTAAGC  GTACGCCAACACAGTGCT |
| mouse β-actin reverse | CGTCATACTCCTGCTTGCTG |
| Human MCSF forward | GAAGGAGGACCAGCAAGTG |
| Human MCSF reverse | GTTCCACCTGTCTGTCATCC |
| Human IL-8 forward | CGGCGACGACCCATTCGA AC |
| Human IL-8 reverse | GAATCGAACCCTGATTCCCCGTC |
| Human RANKL forward | GTCGCCCTGTTCTTCTATTTC |
| Human RANKL reverse | TCTGCTCTGATGTGCTGTG |
| Human IL-6 forward | GACAGCCAACTACGATGATG |
| Human IL-6 reverse | GCAAGTCTCCTCATTGAATCC |
| Human RUNX2 forward | ACCACTCACTACCACACCTAC |
| Human RUNX2 reverse | CTTCCATCAGCGTCAACACC |
| Human MMP13 forward | CCTTGATGCCATTACCAGTC |
| Human MMP13 reverse | AACCTTCCAGAATGTCATAACC |
| Human PTHrP forward | TTACGGCGACGATTCTTCC |
| Human PTHrP reverse | CAGTCACTCCAGAGTCTAACC |
| Human IL-11 forward | ACTGCTGCTGCTGAAGACTC |
| Human IL-11 reverse | CCACCCCTGCTCCTGAAATA |
| Human β-actin forward | CATCCTGCGTCTGGACCT |
| Human β-actin reverse | GTACTTGCGCTCAGGAGGAG |

**Supplementary Table 4. Sequences of primers used for sub-cloning and plasmid construction**

|  | Sequences (5′-3′) |
| --- | --- |
| pri-miR-124 cDNA forward | CATAGAAGATTCTAGCACCCCCATCCCTCTCCC |
| pri-miR-124 cDNA reverse | ATTTAAATTCGAATTGGCAGCCGGAGGGAGCTC |
| psicheck2-IL-11 3′UTR Wild type forward | CCGCTCGAG CCACCGTCCTTCCAAAGCCAGA |
| psicheck2-IL-11 3′UTR Wild type reverse | ATAAGAATGCGGCCGCGCAAAATACACAGTCATGGCAGAAA |
| psicheck2-IL-11 3′UTR Mutant forward | AGCATCGATTTATACTTATTTATTT |
| psicheck2-IL-11 3′UTR Mutant reverse | AGATGCCCCCCAGGCCTCACGGAAG |

|  | | | | | |
| --- | --- | --- | --- | --- | --- |
|  |  | | |  | |
|  |  |  |  |  | |
|  | | | | | |
|  |  |  |  | |  |
|  |  |  |  | |  |
|  | | | | | |
|  |  |  |  | |  |
|  |  |  |  | |  |
|  | | | | | |
|  |  |  |  | |  |
|  |  |  |  | |  |
|  | | | | | |
|  |  |  |  | |  |
|  |  |  |  | |  |
|  |  |  |  | |  |
|  |  |  |  | |  |
|  |  |  |  | |  |
|  | | | | | |
|  |  |  |  | |  |
|  |  |  |  | |  |
|  | | | | | |
|  |  |  |  | |  |
|  |  |  |  | |  |
|  | | | | | |
|  |  |  |  | |  |
|  |  |  |  | |  |
|  | | | | | |
|  |  |  |  | |  |
|  |  |  |  | |  |
|  | | | | | |
|  |  |  |  | |  |
|  |  |  |  | |  |
|  | | | | | |
|  |  |  |  | |  |
|  |  |  |  | |  |
|  | | | | | |
|  |  |  |  | |  |
|  |  |  |  | |  |

|  | | | | | |
| --- | --- | --- | --- | --- | --- |
|  |  | | |  | |
|  |  |  |  |  | |
|  | | | | | |
|  |  |  |  | |  |
|  |  |  |  | |  |
|  | | | | | |
|  |  |  |  | |  |
|  |  |  |  | |  |
|  | | | | | |
|  |  |  |  | |  |
|  |  |  |  | |  |
|  | | | | | |
|  |  |  |  | |  |
|  |  |  |  | |  |
|  |  |  |  | |  |
|  |  |  |  | |  |
|  |  |  |  | |  |
|  | | | | | |
|  |  |  |  | |  |
|  |  |  |  | |  |
|  | | | | | |
|  |  |  |  | |  |
|  |  |  |  | |  |
|  | | | | | |
|  |  |  |  | |  |
|  |  |  |  | |  |
|  | | | | | |
|  |  |  |  | |  |
|  |  |  |  | |  |
|  | | | | | |
|  |  |  |  | |  |
|  |  |  |  | |  |
|  | | | | | |
|  |  |  |  | |  |
|  |  |  |  | |  |
|  | | | | | |
|  |  |  |  | |  |
|  |  |  |  | |  |
